# Cover's universal portfolio, stochastic portfolio theory, and the numéraire portfolio

**DOI:** 10.1111/mafi.12201

**Published:** 2018-10-01

**Authors:** Christa Cuchiero, Walter Schachermayer, Ting‐Kam Leonard Wong

**Affiliations:** ^1^ University of Vienna Vienna Austria; ^2^ Department of Statistical Sciences University of Toronto Toronto Ontario Canada

**Keywords:** Diffusions on the unit simplex, ergodic Markov process, functionally generated portfolios, long‐only portfolios, log‐optimal portfolio, stochastic portfolio theory, universal portfolio

## Abstract

Cover's celebrated theorem states that the long‐run yield of a properly chosen “universal” portfolio is almost as good as that of the best *retrospectively chosen* constant rebalanced portfolio. The “universality” refers to the fact that this result is *model‐free*, that is, not dependent on an underlying stochastic process. We extend Cover's theorem to the setting of stochastic portfolio theory: the market portfolio is taken as the numéraire, and the rebalancing rule need not be constant anymore but may depend on the current state of the stock market. By fixing a stochastic model of the stock market this model‐free result is complemented by a comparison with the numéraire portfolio. Roughly speaking, under appropriate assumptions the asymptotic growth rate coincides for the three approaches mentioned in the title of this paper. We present results in both discrete and continuous time.

## 1 INTRODUCTION

In Fernholz and Karatzas (2009), the question was raised whether there is a relation between Cover's theory of universal portfolio (which appeared as the very first paper of the present journal, see Cover, 1991) and stochastic portfolio theory (SPT henceforth) as initiated by Fernholz (see Fernholz, 2002 and the references therein). After all, both theories ask for general recipes for choosing in a *preference‐free* way good (at least in the long run) portfolios among *d* assets, whose prices over time are given by

$$S=\left(S_{t}^{1},\cdots ,S_{t}^{d}\right).$$

Here, the time *t* varies in T, where T stands either for N={0,1,…} (discrete time) or $R_{+}=\left[0,\infty \right)$ (continuous time). In many cases, *S* is modeled by a stochastic process defined on some probability space. We note, however, that one may also consider a *model‐free* approach where $S={\left(s_{t}^{1},\cdots s_{t}^{d}\right)}_{t\in T}$ is just a deterministic trajectory with values in ${\left(0,\infty \right)}^{d}$. Indeed, Cover and Ordentlich's discrete time results in Cover (1991) and Cover and Ordentlich (1996) are formulated in this model‐free sense. The situation is more subtle in continuous time due to stochastic integration. Jamshidian (1992), extended Cover's universal portfolio to continuous time under a setting of Itô processes satisfying some asymptotic stability conditions.

In SPT, one also seeks robust investment strategies. More precisely, the strategies should be constructed using only observable quantities (such as market weights and their quadratic variations) and should not depend on quantities that are nonobservable or difficult to estimate. In particular, no drift estimation is involved which is usually required in expected utility maximization. These are exactly the principles behind the concept of *functionally generated portfolios* (see Fernholz, 2002, chapter 3). Although in most of the literature an Itô process setting is assumed, much of SPT can be developed in a model‐free setting as done by Pal and Wong (2016) in discrete time and by Schied, Speiser, and Voloshchenko (2016) in continuous time. The reason why it works in continuous time is that the value processes of functionally generated portfolios can be defined without stochastic integration.

In this paper, we connect the two theories and provide additionally a comparison with the numéraire portfolio, which corresponds to the classical log‐optimal portfolio.^1^ Relationships between the two theories were studied in the recent papers by Ichiba and Brod (2014), and Brod (2014) as well as Wong (2015). In particular, Wong (2015) extends Cover's approach to the family of functionally generated portfolios in discrete time and shows that the distribution of wealth in this family satisfies a pathwise large deviation principle.

### 1.1 Summary and discussion of the main results

In this paper, we work under the setting of SPT. Namely, the market portfolio is taken as the benchmark, or “numéraire,” so that the primary assets are the market weights which take values in the open *d*‐simplex defined by $\Delta^{d}=\left\{x\in {\left(0,1\right)}^{d}\;|\;\sum_{i=1}^{d}x^{i}=1\right\}$. Its closure is denoted by ${\bar{\Delta }}^{d}=\left\{x\in {\left[0,1\right]}^{d}\;|\;\sum_{i=1}^{d}x^{i}=1\right\}$. This enables us to analyze strategies which depend on the market weights, and the performance of *relative* wealth with respect to the market portfolio.

#### 1.1.1 Discrete time

We start by summarizing our results in discrete time. We extend Cover's universal portfolio to a class of *M*‐Lipschitz portfolio maps denoted by $L^{M}$. Each element of $L^{M}$ maps the market weights to *long‐only* portfolio weights in ${\bar{\Delta }}^{d}$ (see Definition 3.1).

Denoting by ${\left(V_{t}^{\pi }\right)}_{t=0}^{\infty }$ the *relative wealth process* corresponding to a portfolio strategy^2^ ${\left(\pi_{t}\right)}_{t=1}^{\infty }$, we are interested in comparing the asymptotic growth rates

$$\lim_{T\to \infty }\frac{1}{T}\log\left(V_{T}^{\pi }\right)$$

for certain “optimal” portfolio choices π. More precisely, under suitable conditions we establish asymptotic equality of the growth rates of the following portfolios:

- the *best retrospectively chosen portfolio* at time *T* in the class $L:={⋃}_{M=1}^{\infty }L^{M}$ (in this context $V_{T}^{*,M}$ will denote the relative wealth at time *T* achieved by investing according to the best strategy in $L^{M}$ over the time interval [0, *T*]);
- the *analog of Cover's universal portfolio* whose relative wealth process ${\left(V_{t}\left(\nu \right)\right)}_{t=0}^{\infty }$ is defined in (17) (here ν is a probability measure on L with *full support* on each $L^{M}$);
- the *log‐optimal portfolio* among the class of *long‐only* strategies, whose relative wealth process is denoted by ${\left({\hat{V}}_{t}\right)}_{t=0}^{\infty }$.

The first two portfolios can be compared in a model‐free way (see Theorem 3.9). To compare them with the log‐optimal portfolio, we have to introduce a probabilistic setting. Our main result can then be roughly stated as follows:

Theorem 1.1 Let ${\left(\mu_{t}\right)}_{t=0}^{\infty }$ be a time‐homogenous ergodic Markov process in discrete time describing the dynamics of the market weights. Then

(1) $$\begin{array}{c} \lim_{M\to \infty }\lim_{T\to \infty }\frac{1}{T}\log\left(V_{T}^{*,M}\right)=\lim_{T\to \infty }\frac{1}{T}\log\left(V_{T}\left(\nu \right)\right)=\lim_{T\to \infty }\frac{1}{T}\log\left({\hat{V}}_{T}\right) \end{array}$$

holds almost surely.

Intuitively, this theorem says that a suitable *full support* mixture of strategies (given by the universal portfolio) is asymptotically as good as the best one chosen with hindsight, and the log‐optimal portfolio constructed with full knowledge of the underlying process.

#### 1.1.2 Continuous time

Theorem 1.1, which involves Lipschitz portfolio maps, cannot be extended directly to continuous time because of stochastic integrals. Instead, we consider *functionally generated portfolios* (see Section 4) whose relative wealth processes can be defined in a pathwise manner (see, e.g., Schied et al., 2016). This choice not only allows model‐free considerations but also perfectly connects Cover's theory with SPT in continuous time. By replacing the set $L^{M}$ by certain spaces of functionally generated portfolios and assuming that the log‐optimal portfolio is functionally generated, we get essentially the same theorem as above.

Apart from the work by Jamshidian (1992), universal portfolio theory has only been studied sparingly in continuous time; see, for example, the paper Ichiba, Papathanakos, Banner, Karatzas, and Fernholz (2011) which studied the performance of the universal portfolio under the “Hybrid Atlas” model. To the best of our knowledge, generalizations to nonparametric families of portfolio maps (in continuous time) have not been considered so far. In this sense, our results significantly extend the continuous‐time literature.

Although our approach focuses on the mathematical aspects, universal portfolio strategies have also been studied extensively in an algorithmic framework. See Li and Hoi (2014) for a recent survey and in particular Hazan and Kale (2015).

#### 1.1.3 Discussion of the results

Our model‐free approach has clear advantages over classical ones which heavily rely on a particular model choice. Even in the case when the model class (e.g., the Heston model or Lévy models) is correctly specified, model parameters cannot be estimated precisely and always come with a confidence interval. So, in practice, the *estimated optimal* portfolio is always different from the *true optimal* one. Our results support the idea that a Bayesian average in the spirit of Cover's universal portfolio is, in the long run, better than a suboptimal estimate.

As for the original theorems of Cover and Jamshidian, a valid criticism is of course that we only establish asymptotic equality on a first‐order log‐return basis. As such, a lot of important information is lost in the limit. However, one cannot expect to obtain any information on higher order terms unless further quantitative assumptions are made on the considered models. Cover's aim and also the goal of the present paper is to be as model‐free as possible.^3^ Nevertheless, it is of great theoretical and practical interest to strengthen the asymptotic results to quantitative ones under suitable additional conditions. We hope to address this important question in future research.

The remainder of the paper is organized as follows. In Section 2, we provide a brief overview (in discrete time for convenience) of the main topics of this paper, that is, Cover's theorem, the setting of SPT, and the log‐optimal portfolio. In Section 3, we establish Theorem 1.1 in discrete time (see Theorem 3.10 and Corollary 3.11), whereas Section 4 is dedicated to proving the corresponding statements in continuous time in the setting of functionally generated portfolios and—for the comparison with the log‐optimal portfolio—under the assumption that the market weights follow an ergodic Itô diffusion (see Theorem 4.11 and Corollary 4.13). Some auxiliary and technical proofs are gathered in the Appendix.

## 2 OVERVIEW OF THE THREE PORTFOLIOS

For expositional simplicity, time is discrete in this section.

### 2.1 Cover's universal portfolio

Cover's insight reveals that the “wisdom of hindsight” does not give significant advantages over a properly chosen “universal” portfolio constructed using only historical and current prices of the assets. The relevant optimality criterion here is the asymptotic growth rate of the portfolio.

Let us sketch this—at first glance surprising—result in a particularly easy setting (compare Cover, 1991; Cover & Ordentlich, 1996): Fix T∈N and think of an investor who at time *T* looks back which stock she should have bought at time t=0 (by investing her initial endowment and subsequently holding the stock). There is an obvious solution: pick i∈{1,⋯,d} which maximizes the normalized logarithmic return

(2) $$\begin{array}{c} \frac{1}{T}\left(\log\left(S_{T}^{i}\right)-\log\left(S_{0}^{i}\right)\right). \end{array}$$

The problem with this trading strategy is, of course, that we have to make our choice at time t=0 instead of t=T. Here is the remedy (compare, e.g., Blum & Kalai, 1999): at time t=0 simply divide the initial endowment, say 1€, into *d* portions of $\frac{1}{d}€$, invest each portion in each of the stocks and then hold the resulting portfolio. At time *T*, the normalized logarithmic return satisfies^4^

(3) $$\begin{array}{c} \frac{1}{T}\log\left(V_{T}\right)\geq \frac{1}{T}\log\left(\frac{1}{d}\sum_{j=1}^{d}\frac{S_{T}^{j}}{S_{0}^{j}}\right)\geq \frac{1}{T}\left(\log\left(S_{T}^{i}\right)-\log\left(S_{0}^{i}\right)-\log d\right), \end{array}$$

where again *i* denotes the stock which performed best during the time interval [0, *T*]. Hence, the difference between (2) and (3) can be bounded by $\frac{\log(d)}{T}$ which tends to zero as T→∞. Hence this buy‐and‐hold portfolio, which corresponds to a universal portfolio in the sense of Cover, has asymptotically the same normalized logarithmic return as the—only retrospectively known—best performing stock.

Instead of these “pure” investments, Cover considered a more ambitious setting, namely, all *constant rebalanced portfolio strategies*: let $b=\left(b^{1},\cdots ,b^{d}\right)\in {\bar{\Delta }}^{d}$, that is, $b^{j}\geq 0$ and $\sum_{j=1}^{d}b^{j}=1$. The value of the corresponding constant rebalanced portfolio ${\left(V_{t}\left(b\right)\right)}_{t=0}^{\infty }$ starting at $V_{0}\left(b\right)=1$ is defined by holding throughout the proportion $b^{j}$ of the current wealth in stock *j*, so that $V_{0}\left(b\right)=1$ and

(4) $$\begin{array}{c} \frac{V_{t+1}\left(b\right)}{V_{t}\left(b\right)}\left(s\right)=\sum_{j=1}^{d}b^{j}\frac{s_{t+1}^{j}}{s_{t}^{j}} \end{array}$$

for each trajectory $s={\left({\left(s_{t}^{j}\right)}_{j=1}^{d}\right)}_{t=0}^{\infty }\subset {\left(0,\infty \right)}^{d}$ of the stocks.

Fix again *T* and define the quantity $V_{T}^{*}$ by

(5) $$\begin{array}{c} V_{T}^{*}\left(s\right)=\max_{b\in {\bar{\Delta }}^{d}}V_{T}\left(b\right)\left(s\right), \end{array}$$

which is a function of the trajectory $s={\left(s_{t}^{1},\cdots ,s_{t}^{d}\right)}_{t=0}^{T}$. Again, the idea is that, with hindsight, that is, knowing ${\left(s_{t}^{1},\cdots ,s_{t}^{d}\right)}_{t=0}^{T}$, one considers the best weight $b\in {\bar{\Delta }}^{d}$ which attains the maximum (5). Cover's goal is to construct a portfolio which generates wealth that performs asymptotically as well as the process ${\left(V_{T}^{*}\right)}_{T=0}^{\infty }$ as T→∞, uniformly over all price paths. For this reason, the portfolio is said to be *universal*. In order to do so, let ν be a probability measure on ${\bar{\Delta }}^{d}$ which replaces the previous uniform distribution over the *d* stocks. The universal portfolio is built by investing at time 0 the portion dν(b) of initial capital in the constant rebalanced portfolio V(b) and by subsequently following the constant rebalanced portfolio process ${\left(V_{t}\left(b\right)\right)}_{t=0}^{T}$. The explicit formula for the wealth is

(6) $$\begin{array}{c} V_{t}\left(\nu \right)\left(s\right)=\int_{{\bar{\Delta }}^{d}}V_{t}\left(b\right)\left(s\right)d\nu \left(b\right), \end{array}$$

where $V_{t}\left(b\right)$ is defined by (4). The portfolio weight of the corresponding *universal portfolio* is given by the wealth‐weighted average

(7) $$\begin{array}{c} b_{t}^{\nu }\left(s\right)=\frac{\int_{{\bar{\Delta }}^{d}}bV_{t}\left(b\right)\left(s\right)d\nu \left(b\right)}{\int_{{\bar{\Delta }}^{d}}V_{t}\left(b\right)\left(s\right)d\nu \left(b\right)}. \end{array}$$

Let us now recall Cover's celebrated result:

Theorem 2.1 (Cover, 1991): Let ν be a probability measure on ${\bar{\Delta }}^{d}$ with full support. Then

(8) $$\begin{array}{c} \lim_{T\to \infty }\frac{1}{T}\left(\log\left(V_{T}\left(\nu \right)\left(s\right)\right)-\log\left(V_{T}^{*}\left(s\right)\right)\right)=0 \end{array}$$

for *all* trajectories $s={\left(s_{t}^{1},\cdots ,s_{t}^{d}\right)}_{t=0}^{\infty }$ for which there are constants 0<c≤C<∞ such that

(9) $$\begin{array}{c} c\leq \frac{s_{t+1}^{j}}{s_{t}^{j}}\leq C,\;\text{for}\;\text{all}\;j=1,\cdots ,d\;\text{and}\;\text{all}\;t\in N. \end{array}$$

The proof is given in the Appendix.

Remark 2.2 As shown by Cover and Ordentlich (1996), the condition (9) can be dropped at least when ν is the uniform or Dirichlet $\left(\frac{1}{2},\cdots ,\frac{1}{2}\right)$ distribution on $\Delta^{d}$ (see also Blum & Kalai, 1999, for an elegant proof in case of the uniform distribution).

Remark 2.3 Let $M_{1}\left({\bar{\Delta }}^{d}\right)$ be the set of probability measures on ${\bar{\Delta }}^{d}$. For each $\mu \in M_{1}\left({\bar{\Delta }}^{d}\right)$, consider the value $\int_{{\bar{\Delta }}^{d}}V_{T}\left(b\right)\left(s\right)d\mu \left(b\right)$ of the mixture portfolio with initial measure μ. Note that the constant rebalanced portfolio $V_{T}\left(b\right)$ corresponds to the case where μ is the point mass at *b*. It is easy to see that

$$\underset{\mu \in M_{1}\left(\bar{\Delta }\right)}{\sup}\int_{{\bar{\Delta }}^{d}}V_{T}\left(b\right)\left(s\right)d\mu \left(b\right)=V_{T}^{*}\left(s\right),$$

where $V_{T}^{*}\left(s\right)$ is defined by (5). It follows that the universal portfolio (6) (with initial measure ν) is still asymptotically optimal in the larger class

(10) $$\begin{array}{c} \left\{{\left(\frac{\int_{{\bar{\Delta }}^{d}}bV_{t}\left(b\right)d\mu \left(b\right)}{\int_{{\bar{\Delta }}^{d}}V_{t}\left(b\right)\left(s\right)d\mu \left(b\right)}\right)}_{t\geq 0}\;|\;\mu \in M_{1}\left({\bar{\Delta }}^{d}\right)\right\}. \end{array}$$

### 2.2 SPT, portfolio maps, and the corresponding universal portfolio

In SPT, we let $\left(s^{1},\cdots ,s^{d}\right)$ denote the market capitalizations of the stocks rather than their prices. Then we define the vector of *market weights* $\left(\mu^{1},\cdots ,\mu^{d}\right)\in \Delta^{d}$ by

$$\begin{array}{c} \left(\mu^{1},\cdots ,\mu^{d}\right)=\left(\frac{s^{1}}{s^{1}+\cdots +s^{d}},\cdots ,\frac{s^{d}}{s^{1}+\cdots +s^{d}}\right). \end{array}$$

This amounts to taking the *market portfolio* (whose value at time *t* is $\sum_{j=1}^{d}s_{t}^{j}$) as the *numéraire* (compare Delbaen & Schachermayer, 1995 and Fernholz & Karatzas, 2010a).

The *relative wealth process* ${\left(V_{t}^{\pi }\right)}_{t=0}^{\infty }$, expressed in units of the market portfolio and starting at $V_{0}=1$, is obtained by the following recursive relation:^5^

(11) $$\begin{array}{c} \frac{V_{t+1}^{\pi }}{V_{t}^{\pi }}=\sum_{j=1}^{d}\pi_{t+1}^{j}\frac{\mu_{t+1}^{j}}{\mu_{t}^{j}}. \end{array}$$

In general, we allow all predictable, admissible trading strategies ${\left(\pi_{t}\right)}_{t=1}^{\infty }$, where the portfolio weight $\pi_{t}$ is used over the time interval [t−1,t]. In this paper, all trading strategies are fully invested in the equity market, that is, the portfolio weights sum to 1 for all *t*. In particular, the strategies do not lend or borrow money. Henceforth, all wealth processes are measured in units of the market portfolio.

We will focus on trading strategies defined by (deterministic) *portfolio maps*. These are (Borel) measurable functions

(12) $$\begin{array}{c} \pi :\Delta^{d}\to {\bar{\Delta }}^{d}, \end{array}$$

which associate to the current market capitalization $\mu_{t}=\left(\mu_{t}^{1},\cdots ,\mu_{t}^{d}\right)$ the weights $(\pi \left(\mu_{t}\right)=\left(\pi^{1}\left(\mu_{t}\right),\cdots ,\pi^{d}\left(\mu_{t}\right)\right)$ according to which an agent distributes current wealth among the *d* stocks at time *t*. The *constant rebalanced portfolio strategies* considered by Cover correspond to the constant functions $\pi :\Delta^{d}\to {\bar{\Delta }}^{d}$.

In this paper, we extend Cover's theory of *constant rebalanced portfolios* to certain families of *portfolio maps*. First, we note that Cover's and Jamshidian's definition of a universal portfolio as in (7) and (6) can be easily extended to a general setting. Let G denote some appropriate space of portfolio maps, B(G) its Borel σ‐algebra and ν some probability measure on G.

Definition 2.4 Let ν be a probability measure on (G,B(G)). Then, the corresponding universal portfolio at time *t* is given by the wealth‐weighted average

(13) $$\begin{array}{c} \pi_{t}^{\nu }=\frac{\int_{G}\pi V_{t}^{\pi }d\nu \left(\pi \right)}{\int_{G}V_{t}^{\pi }d\nu \left(\pi \right)}. \end{array}$$

From (11), it is easily seen that the wealth generated by $\pi^{\nu }$ is given by

(14) $$\begin{array}{c} V_{T}\left(\nu \right)=\int_{G}V_{T}^{\pi }d\nu \left(\pi \right). \end{array}$$

### 2.3 The log‐optimal portfolio

To define the log‐optimal portfolio, we consider a probabilistic setting. The stock price process $S={\left(S_{t}^{1},\cdots ,S_{t}^{d}\right)}_{t=0}^{\infty }$ and the corresponding relative market capitalizations $\mu ={\left(\mu_{t}^{1},\cdots ,\mu_{t}^{d}\right)}_{t=0}^{\infty }$ are now assumed to be stochastic processes defined on a filtered probability space $\left(\Omega ,F,{\left(F_{t}\right)}_{t=0}^{\infty },P\right)$.

There is a large literature on the log‐optimal portfolio (see, e.g., Becherer, 2001; Karatzas & Kardaras, 2007 and the references given there). For a fixed horizon *T*, this portfolio is by definition the maximizer of the expected logarithmic growth rate

(15) $$\begin{array}{c} E\left[\log\left(V_{T}^{\pi }\right)\right]=E\left[\sum_{t=0}^{T-1}\log\left(\sum_{j=1}^{d}\pi_{t+1}^{j}\frac{\mu_{t+1}^{j}}{\mu_{t}^{j}}\right)\right] \end{array}$$

over all predictable, admissible trading strategies ${\left(\pi_{t}\right)}_{t=1}^{T}$. Under mild assumptions on the process a unique optimizer exists; see, for example, Becherer (2001) and Kramkov and Schachermayer (1999).

To connect the log‐optimal portfolio with universal portfolios in the sense of Definition 2.4, we need appropriate assumptions. We will assume that μ is a time‐homogenous Markov process, and we will restrict to *long‐only* portfolios in the optimization of (15). These imply that the optimal portfolio in (15) (over the set of predictable processes taking values in ${\bar{\Delta }}^{d}$) has the form $\pi_{t}=\pi \left(\mu_{t-1}\right)$, where $\pi :\Delta^{d}\mapsto {\bar{\Delta }}^{d}$ as in (12). We denote the corresponding optimizer by $\hat{\pi }$.

The Markovian assumption can be motivated by the stability of capital distributions of equity markets (see Fernholz, 2002, chapter 5). In SPT, this led to systems of interacting Brownian particles whose dynamics depend on their relative rankings. Under suitable conditions, these systems show behaviors observed in large equity markets. See, for example, Banner, Fernholz, and Karatzas (2005) and Ichiba et al. (2011) for “Atlas”‐type models and the references therein.^6^ We also refer to Kardaras and Robertson (2012) which studies the growth optimal portfolio in a Markovian setting with uncertainties.

## 3 A COMPARISON OF THE THREE APPROACHES—THE DISCRETE TIME CASE

Throughout this section, we work in discrete time and assume that the market weights are described by a *d*‐dimensional path $\mu ={\left(\mu_{t}\right)}_{t=0}^{\infty }$ with values in $\Delta^{d}$. We consider as far as possible a model‐free approach, but will introduce a probabilistic setting when the log‐optimal portfolio is involved.

### 3.1 Definitions of the portfolios

We start by defining rigorously, in the present setting, the three portfolios introduced in Sections 1 and 2.

#### 3.1.1 The best retrospectively chosen portfolio

Consider Cover's theme of choosing retrospectively at time *T* a strategy which is optimal within a certain class of strategies, in our case portfolio maps $\pi :\Delta^{d}\to {\bar{\Delta }}^{d}$. A moment's reflection reveals that it does not make sense to allow to choose among *all measurable* functions $\pi :\Delta^{d}\to {\bar{\Delta }}^{d}$. Indeed, there is no restriction to choose π such that $\pi \left(\mu_{t}\right)=e_{j(t)}$, where j(t)∈{1,⋯,d} maximizes $\mu_{t+1}^{j}/\mu_{t}^{j}$. This is asking for too much clairvoyance and does not allow for meaningful results (compare Cover & Ordentlich, 1996; and Blum & Kalai, 1999, section 5).

However, it does make sense (economically as well as mathematically) to restrict to more regular trading strategies. In particular, we work with the following set of *M*‐Lipschitz portfolio maps. For ε>0, we let ${\bar{\Delta }}_{\varepsilon }^{d}$ denote the set of $x\in \Delta^{d}$ satisfying $x^{j}\geq \frac{\varepsilon }{d}$, for j=1,⋯,d. Also we let ${∥\cdot ∥}_{1}$ be the usual 1‐norm.

Definition 3.1 For M>0, we denote by $L^{M}$ the set of all *M*‐Lipschitz functions $\Delta^{d}\to {\bar{\Delta }}_{M^{-1}}^{d}$, that is, ${∥\pi \left(x\right)-\pi \left(y\right)∥}_{1}\leq L{∥x-y∥}_{1}$, $x,y\in \Delta^{d}$.

Remark 3.2 The set $L^{M}$ of *M*‐Lipschitz functions $\pi :\Delta^{d}\to {\bar{\Delta }}_{M^{-1}}^{d}$ is a compact metric space with respect to the topology of uniform convergence induced by the norm ${∥\pi ∥}_{\infty }={\sup\{∥\pi \left(x\right)∥}_{1}:x\in \Delta^{d}\}$.

Remark 3.3 Instead of Lipschitz functions we could just as well consider other compact function spaces, for example, Hölder spaces equipped with a proper norm. This is done in the context of functionally generated portfolios in Section 4.

The retrospectively chosen best performing portfolio among the above Lipschitz maps is defined as follows:

Definition 3.4 For a given trajectory ${\left(\mu_{t}\right)}_{t=0}^{T}\in {\left(\Delta^{d}\right)}^{T+1}$, we define

(16) $$\begin{array}{c} V_{T}^{*,M}=\underset{\pi \in L^{M}}{\sup}V_{T}^{\pi }=\underset{\pi \in L^{M}}{\sup}\prod_{t=0}^{T-1}\left(\sum_{j=1}^{d}\pi^{j}\left(\mu_{t}\right)\frac{\mu_{t+1}^{j}}{\mu_{t}^{j}}\right). \end{array}$$

By compactness (see Remark 3.2) and continuity of the map $\pi \mapsto V_{T}^{\pi }$, there exists an optimizer $\pi^{*,M}\in L^{M}$ (not necessarily unique) such that $V_{T}^{*,M}=V_{T}^{\pi^{*,M}}$, thus the sup above can be replaced by max .

#### 3.1.2 The universal portfolio

Our aim is to find a *predictable* process $\pi^{M}={\left(\pi_{t}^{M}\right)}_{t=1}^{\infty }$, that is, one which depends only on the history of the market weights, such that the performance of ${\left(V_{t}^{\pi^{M}}\right)}_{t=0}^{\infty }$ is asymptotically as good as that of ${\left(V_{t}^{*,M}\right)}_{t=0}^{\infty }$. This can be achieved by the universal portfolio introduced in Definition 2.4, where the G is now $L^{M}$ as in Definition 3.1. As $L^{M}$ is a compact metric space, we may find a (Borel) probability measure ν on $\left(L_{M},∥\cdot {∥}_{\infty }\right)$ with full support; this will be essential for establishing an analog to Theorem 2.1. The (relative) wealth of the universal portfolio is given, as in (14), by

(17) $$\begin{array}{c} V_{T}^{M}\left(\nu \right)=\int_{L^{M}}V_{T}^{\pi }d\nu \left(\pi \right). \end{array}$$

#### 3.1.3 The log‐optimal portfolio

In order to relate the universal portfolio to the (long‐only) log‐optimal portfolio, we assume that $\mu ={\left(\mu_{t}\right)}_{t=0}^{\infty }$ is a time‐homogeneous Markov process (see Section 2.3). Here is a precise statement.

Assumption 3.5 The process μ is a time homogeneous, ergodic Markov process with a unique invariant measure ϱ on the open simplex $\Delta^{d}$.

We denote the transition kernel of the chain by ${\left(\varrho \left(x,\cdot \right)\right)}_{x\in \Delta^{d}}$, that is, for all Borel sets $A\subseteq {\bar{\Delta }}^{d}$ we have $P\left[\mu_{t+1}\in A|F_{t}\right]=\varrho \left(\mu_{t},A\right)$. For further notions concerning ergodic Markov processes, we refer to Eberle (2016).

The *long‐only log‐optimal* trading strategy $\hat{\pi }$, as noted above, is given in terms of a portfolio map. Given that $\mu_{t}=x\in \Delta^{d}$, we know the conditional law ϱ(x,·) of $\mu_{t+1}$. We therefore choose $\hat{\pi }\left(x\right)\in {\bar{\Delta }}^{d}$ as the maximizer

(18) $$\hat{\pi }\left(x\right)=\underset{p\in {\bar{\Delta }}^{d}}{arg max}\left(\int_{\Delta^{d}}\log\left(〈p,\frac{y}{x}〉\right)\varrho \left(x,dy\right)\right)$$

and assume that $\hat{\pi }\left(\cdot \right)$ can be chosen to be measurable (here ⟨,⟩ denotes the Euclidean dot product). For $x\in \Delta^{d}$, define the number L(x) as the value of the optimization problem (18), that is,

(19) $$L\left(x\right)=\max_{p\in {\bar{\Delta }}^{d}}\left(\int_{\Delta_{d}}\log\left(〈p,\frac{y}{x}〉\right)\varrho \left(x,dy\right)\right)=\int_{\Delta_{d}}\log\left(〈\hat{\pi }\left(x\right),\frac{y}{x}〉\right)\varrho \left(x,dy\right).$$

Considering π(x)=x (which corresponds to the market portfolio) we clearly have L(x)≥0 for each $x\in \Delta^{d}$. We obtain the a.s. relation

$$\begin{array}{c} L\left(x\right)=E\left[\left.\log\left(\frac{{\hat{V}}_{t+1}}{{\hat{V}}_{t}}\right)\right|\mu_{t}=x\right], \end{array}$$

where $\hat{V}={\left({\hat{V}}_{t}\right)}_{t=0}^{\infty }$ denotes the long‐only log‐optimal wealth process $V^{\hat{\pi }}$ defined by the portfolio map $\hat{\pi }$ via (11).

Assumption 3.6 Using the above notation we assume that

(20) $$\begin{array}{c} L:=\int_{\Delta^{d}}L\left(x\right)d\varrho \left(x\right)<\infty . \end{array}$$

Applying Birkhoff's ergodic theorem for discrete time Markov processes (see Eberle, 2016, theorem 2.2, section 2.1.4), we have the following result.

Theorem 3.7 Under Assumptions 3.5 and 3.6, we have that, for ϱ‐a.e. starting value $\mu_{0}\in \Delta^{d}$,

(21) $$\lim_{T\to \infty }\frac{1}{T}\log\left({\hat{V}}_{T}\right)=L,$$

the limit holding true a.s. as well as in *L* ^1^.

More generally, let $\pi :\Delta^{d}\to {\bar{\Delta }}^{d}$ be any measurable portfolio map such that

(22) $$L^{\pi }:=\int_{\Delta^{d}}\left(\int_{\Delta^{d}}\log\left(〈\pi \left(x\right),\frac{y}{x}〉\right)\varrho \left(x,dy\right)\right)d\varrho \left(x\right)>-\infty .$$

We then have, for ϱ‐a.s. starting value μ_0_, that

(23) $$\lim_{T\to \infty }\frac{1}{T}\log\left(V_{T}^{\pi }\right)=L^{\pi }$$

a.s. as well as in *L* ^1^.

In general, there is little reason why the function $\hat{\pi }$ should have better regularity properties than being just measurable. On the other hand, we may approximate $\hat{\pi }$ by more regular functions, in particular by functions in $L^{M}$. This will be crucial for comparing the asymptotic growth rates. The following result is intuitively obvious, but the proof turns out to be quite technical and will be given in the Appendix.

Lemma 3.8 Under Assumptions 3.5 and 3.6, for any ε>0 there exist M>0 and an *M* *‐Lipschitz function* $\pi_{Lip}\in L^{M}$ such that

$$L^{\pi_{Lip}}>L-\varepsilon ,$$

where *L* and $L^{\pi }$ are given in (20) and (22), respectively. In particular, we have $L=\sup_{M}\sup_{\pi \in L^{M}}L^{\pi }$.

### 3.2 Asymptotically equivalent growth rates

We are now ready to compare the asymptotic performance of the three approaches. We first establish an analog of Theorem 2.1.

Theorem 3.9 Fix M>0 and a Borel probability measure ν with full support on $L^{M}$. For every trajectory ${\left(\mu_{t}\right)}_{t=0}^{\infty }$ in $\Delta^{d}$, we have

(24) $$\begin{array}{c} \lim_{T\to \infty }\frac{1}{T}\left(\log\left(V_{T}^{*,M}\right)-\log\left(V_{T}^{M}\left(\nu \right)\right)\right)=0. \end{array}$$

The inequality “≥” is obvious. For the reverse inequality, we follow the argument of Blum and Kalai (1999). As $L^{M}$ is compact and ν has full support, it is not difficult to see that for any η>0, there exists δ>0 such that every η‐neighborhood of a point $\pi \in L^{M}$ has ν‐measure bigger than δ.

Let a trajectory ${\left(\mu_{t}\right)}_{t=0}^{\infty }$ in $\Delta^{d}$ be given. For a fixed time *T*, let $\pi^{*,M}\in L^{M}$ be an optimizer of (16). Consider a portfolio map $\pi^{M}\in L^{M}$ with $∥\pi^{M}-\pi^{*,M}{∥}_{\infty }<\eta$, that is, such that, for every $x\in \Delta^{d}$ we have $∥\pi^{M}\left(x\right)-\pi^{*,M}{\left(x\right)∥}_{1}=\sum_{j=1}^{d}\left|\pi^{M}{\left(x\right)}^{j}-\pi^{*,M}{\left(x\right)}^{j}\right|<\eta$.

Choose η>0 small enough so that α=ηMd<1 and define, for $x\in \Delta^{d}$,

(25) $$\begin{array}{c} \tilde{\pi }\left(x\right)=\frac{1}{\alpha }\pi^{M}\left(x\right)-\frac{1-\alpha }{\alpha }\pi^{*,M}\left(x\right). \end{array}$$

Rearranging, we have

(26) $$\begin{array}{c} \pi^{M}\left(x\right)=\left(1-\alpha \right)\pi^{*,M}\left(x\right)+\alpha \tilde{\pi }\left(x\right). \end{array}$$

It is easy to see that $\tilde{\pi }$ maps $\Delta^{d}$ into ${\bar{\Delta }}^{d}$.

Using (26), we have the estimate

(27) $$\begin{array}{ccc} \frac{1}{T}\log V_{T}^{\pi^{M}} & = & \frac{1}{T}\sum_{t=0}^{T-1}\log\left(〈\pi^{M}\left(\mu_{t}\right),\frac{\mu_{t+1}}{\mu_{t}}〉\right)\\ & \geq & \frac{1}{T}\sum_{t=0}^{T-1}\log\left(〈\left(1-\alpha \right)\pi^{*,M}\left(\mu_{t}\right),\frac{\mu_{t+1}}{\mu_{t}}〉\right)\\ & = & \frac{1}{T}\log\left(V_{T}^{*,M}\right)+\log\left(1-\alpha \right). \end{array}$$

Fix ε>0. Choosing η>0 sufficiently small we can make α=ηMd small enough such that the final term is bigger than −ε. Summing up, we have

(28) $$\begin{array}{c} \frac{1}{T}\left[\log\left(V_{T}^{*,M}\right)-\log\left(V_{T}^{\pi^{M}}\right)\right]<\varepsilon \end{array}$$

whenever $∥\pi^{M}-\pi^{*,M}{∥}_{\infty }<\eta$.

Denote by $B=B_{\eta }\left(\pi^{*,M}\right)$ the ${∥\cdot ∥}_{\infty }$‐ball with radius η in $L^{M}$ which has ν‐measure at least δ>0, where δ only depends on η. As each element $\pi^{M}$ of *B* satisfies (28) we have

(29) $$\begin{array}{c} \frac{1}{T}\log\left(V_{T}^{M}\left(\nu \right)\right)\geq \frac{\log(\delta )}{T}+\frac{1}{T}\log\left(V_{T}^{*,M}\right)-\varepsilon . \end{array}$$

Now (24) is proved by sending in (29) *T* to infinity and letting ε to zero. □

Note that in Theorem 3.9 we do not need the uniform boundedness condition (9) (compare this result with Wong, 2015, lemma 3.3). We now combine Lemma 3.8 (which is probabilistic) with Theorem 3.9 (which is pathwise) to obtain—under suitable assumptions–equality of the asymptotic performance among the three portfolios. We first consider the space $L^{M}$ for a fixed *M*. In Corollary 3.11, we then formulate a result for $L={⋃}_{M}L^{M}$.

Theorem 3.10 Let $\Omega ={\left(\Delta^{d}\right)}^{N}$ be the canonical path space equipped with its natural filtration and a probability measure P. Define $\mu ={\left(\mu_{t}\right)}_{t=0}^{\infty }$ to be the canonical process, that is, $\mu_{t}\left(\omega \right)=\omega_{t}$, which takes values in $\Delta^{d}$ and satisfies Assumptions 3.5 and 3.6. Moreover, let M>0 be a fixed Lipschitz constant for the space $L^{M}$. Consider the following objects that are defined for each trajectory ${\left(\mu_{t}\right)}_{t=0}^{\infty }$:^7^

(i) Define for each T∈N the portfolio map $\pi^{*,M}\in L^{M}$ as well as the corresponding wealth $V_{T}^{*,M}:=V_{T}^{\pi^{*,M}}$ as in (16) .
(ii) Fix a probability measure ν on $L^{M}$ with full support and consider the wealth process of the universal portfolio ${\left(V_{t}^{M}\left(\nu \right)\right)}_{t=0}^{\infty }$ as of (17).
(iii) Define the log‐optimal portfolio among the portfolio maps $\pi \in L^{M}$ by
  (30) $$\begin{array}{c} {\hat{\pi }}^{M}=\underset{\pi \in L^{M}}{arg max}\int_{\Delta^{d}}\left[\int_{\Delta^{d}}\log\left(〈\pi \left(x\right),\frac{y}{x}〉\right)\varrho \left(x,dy\right)\right]d\varrho \left(x\right) \end{array}$$
  and the corresponding wealth process ${\left({\hat{V}}_{t}^{M}\right)}_{t=0}^{\infty }={\left(V_{t}^{{\hat{\pi }}^{M}}\right)}_{t=0}^{\infty }$ via (11).

Then, we have P‐a.s.

(31) $$\begin{array}{c} \underset{T\to \infty }{lim inf}\frac{1}{T}\log\left(V_{T}^{*,M}\right)=\underset{T\to \infty }{lim inf}\frac{1}{T}\log\left(V_{T}^{M}\left(\nu \right)\right)=\lim_{T\to \infty }\frac{1}{T}\log\left({\hat{V}}_{T}^{M}\right)=\underset{\pi \in L^{M}}{\sup}L^{\pi }, \end{array}$$

where $L^{\pi }$ is given in (22). In addition, the first equality holds for *all* trajectories ${\left(\mu_{t}\right)}_{t=0}^{\infty }$ in $\Delta^{d}$.

We first note that ${\hat{\pi }}^{M}$ is well‐defined; simply use the compactness of $L^{M}$ with respect to ${∥\cdot ∥}_{\infty }$ (compare the proof of Lemma 3.8). Note also that by the ergodic theorem (Theorem 3.7), we have for each $\pi \in L^{M}$

$$\lim_{T\to \infty }\frac{1}{T}\log V_{T}^{\pi }=L^{\pi }\;P\text{-a.s.},$$

where $L^{\pi }$ is defined by (22). In particular, as ${\hat{\pi }}^{M}\in L^{M}$ by definition, we have

(32) $$\begin{array}{c} \lim_{T\to \infty }\frac{1}{T}\log{\hat{V}}_{T}^{M}=\underset{\pi \in L^{M}}{\sup}L^{\pi }\;P\text{-a.s.} \end{array}$$

That the first equality in (31) holds for *all* trajectories ${\left(\mu_{t}\right)}_{t=0}^{\infty }$ in $\Delta^{d}$ was shown in Theorem 3.9.

For each fixed T∈N, we obviously have

(33) $$\begin{array}{c} \frac{1}{T}\log\left({\hat{V}}_{T}^{M}\right)\leq \frac{1}{T}\log\left(V_{T}^{*,M}\right)\;P\text{-a.s.}\; \end{array}$$

Using (32), (33), and Theorem 3.9 we thus have P‐a.s.

(34) $$\begin{array}{c} \underset{\pi \in L^{M}}{\sup}L^{\pi }=\lim_{T\to \infty }\frac{1}{T}\log\left({\hat{V}}_{T}^{M}\right)\leq \underset{T\to \infty }{lim inf}\frac{1}{T}\log\left(V_{T}^{*,M}\right)=\underset{T\to \infty }{lim inf}\frac{1}{T}\log\left(V_{T}^{M}\left(\nu \right)\right). \end{array}$$

On the other hand, by the definition of ${\left({\hat{V}}_{t}^{M}\right)}_{t=0}^{\infty }$ as the log‐optimizer within the class $L^{M}$, we have

(35) $$\begin{array}{c} E\left[\log\left(V_{T}^{M}\left(\nu \right)\right)\right]\leq \underset{\pi \in L^{M}}{\sup}E\left[\log\left(V_{T}^{\pi }\right)\right]=E\left[\log\left({\hat{V}}_{T}^{M}\right)\right]. \end{array}$$

To see this, note that the universal portfolio is given by (13). By the time‐homogenous Markovianity it is thus sufficient to dominate the left‐hand side of (35) by taking the supremum over elements in $L^{M}$.

Combining now (35), Theorem 3.7 and (34) yields that

$$\begin{array}{cc} E\left[\underset{T\to \infty }{lim inf}\frac{1}{T}\log\left(V_{T}^{M}\left(\nu \right)\right)\right] & \leq \underset{T\to \infty }{lim inf}\frac{1}{T}E\left[\log V_{T}^{M}\left(\nu \right)\right]\\ & \leq \lim_{T\to \infty }\frac{1}{T}E\left[\log\left({\hat{V}}_{T}^{M}\right)\right]\\ & =\lim_{T\to \infty }\frac{1}{T}\log\left({\hat{V}}_{T}^{M}\right)\\ & \leq \underset{T\to \infty }{lim inf}\frac{1}{T}\log V_{T}^{*,M}\\ & =\underset{T\to \infty }{lim inf}\frac{1}{T}\log\left(V_{T}^{M}\left(\nu \right)\right),\;P\text{-a.s.} \end{array}$$

Here, the first inequality follows from Fatou's lemma (note here that $\frac{1}{T}\log\left(V_{T}^{M}\left(\nu \right)\right)$ is bounded from below, see, e.g., (29)). From this we see that the quantity ${lim inf}_{T\to \infty }\frac{1}{T}\log\left(V_{T}^{M}\left(\nu \right)\right)$ is P‐a.s. constant and equal to $\lim_{T\to \infty }\frac{1}{T}\log\left({\hat{V}}_{T}^{M}\right)$. This completes the proof of the theorem. □

Next we will send *M* to infinity in the following way. For M=1,2,3,⋯ choose a measure $\nu^{M}$ on $L^{M}$ with full support. Define $\nu =\sum_{M=1}^{\infty }2^{-M}\nu^{M}$ and the wealth of the universal portfolio V(ν) as in (17) by

(36) $$\begin{array}{c} V_{t}\left(\nu \right)=\int_{L}V_{t}^{\pi }d\nu \left(\pi \right),\;t\in N. \end{array}$$

where $L={⋃}_{M=1}^{\infty }L^{M}$. Recall that ${\left({\hat{V}}_{t}\right)}_{t=0}^{\infty }$ is the wealth process of the (long‐only) log‐optimal portfolio (18).

Corollary 3.11 Under the assumptions of Theorem 3.10 we have P‐a.s.

(37) $$\begin{array}{c} \lim_{M\to \infty }\lim_{T\to \infty }\frac{1}{T}\log V_{T}^{*,M}=\lim_{T\to \infty }\frac{1}{T}\log V_{T}\left(\nu \right)=\lim_{T\to \infty }\frac{1}{T}\log{\hat{V}}_{T}=L, \end{array}$$

where *L* is defined in (20).

Letting M→∞ in (31), we have

$$\lim_{M\to \infty }\underset{T\to \infty }{lim inf}\frac{1}{T}\log V_{T}^{*,M}=\lim_{M\to \infty }\underset{\pi \in L^{M}}{\sup}L^{\pi }=L=\lim_{T\to \infty }\frac{1}{T}\log{\hat{V}}_{T},$$

where the last equality follows from Theorem 3.7 and the second last follows from Lemma 3.8. By construction $V_{T}\left(\nu \right)\geq 2^{-M}V_{T}^{M}\left(\nu^{M}\right)$ for every *M*, so we have by Theorem 3.9 for every *M*

$$\underset{T\to \infty }{lim inf}\frac{1}{T}\log V_{T}\left(\nu \right)\geq \underset{T\to \infty }{lim inf}\frac{1}{T}\left(-M\log2+\log V_{T}^{M}\left(\nu^{M}\right)\right)=\underset{T\to \infty }{lim inf}\frac{1}{T}\log V_{T}^{*,M},$$

and hence also

$$\underset{T\to \infty }{lim inf}\frac{1}{T}\log V_{T}\left(\nu \right)\geq \lim_{M\to \infty }\underset{T\to \infty }{lim inf}\frac{1}{T}\log V_{T}^{*,M}.$$

Using the same argument as in the last part of the proof of Theorem 3.10, we get

(38) $$\begin{array}{c} \lim_{M\to \infty }\underset{T\to \infty }{lim inf}\frac{1}{T}\log V_{T}^{*,M}=\underset{T\to \infty }{lim inf}\frac{1}{T}\log V_{T}\left(\nu \right)=\lim_{T\to \infty }\frac{1}{T}\log{\hat{V}}_{T}=L. \end{array}$$

Now the corollary is proved if

(39) $$\begin{array}{c} \underset{T\to \infty }{lim sup}\frac{1}{T}\left(\log V_{T}\left(\nu \right)-\log{\hat{V}}_{T}\right)=\underset{T\to \infty }{lim sup}\frac{1}{T}\log\left(\frac{V_{T}\left(\nu \right)}{{\hat{V}}_{T}}\right)=0\; \end{array}$$

holds P‐a.s. As by Lemma 3.12, ${\left(\frac{V_{t}\left(\nu \right)}{{\hat{V}}_{t}}\right)}_{t=0}^{\infty }$ is a nonnegative supermartingale, it converges P‐a.s. to a finite limit as t→∞. This in turn implies (39) and proves the assertion. □

Lemma 3.12 The process ${\left(\frac{V_{t}\left(\nu \right)}{{\hat{V}}_{t}}\right)}_{t=0}^{\infty }$ is a nonnegative supermartingale.

First note that for any $\pi :\Delta^{d}\to {\bar{\Delta }}^{d}$, ${\left(\frac{V_{t}^{\pi }}{{\hat{V}}_{t}}\right)}_{t=0}^{\infty }$ is a nonnegative supermartingale. Indeed, by Lemma 3.13 we have

$$\begin{array}{c} E\left[\frac{V_{t+1}^{\pi }}{{\hat{V}}_{t+1}}|F_{t}\right]=\frac{V_{t}^{\pi }}{{\hat{V}}_{t}}\int_{\Delta^{d}}\frac{\left\langle \pi \left(\mu_{t}\right),\frac{y}{\mu_{t}}\right\rangle }{\left\langle \hat{\pi }\left(\mu_{t}\right),\frac{y}{\mu_{t}}\right\rangle }\varrho \left(\mu_{t},dy\right)\leq \frac{V_{t}^{\pi }}{{\hat{V}}_{t}}. \end{array}$$

By Fubini's theorem, we get the supermartingale property of ${\left(\frac{V_{t}\left(\nu \right)}{{\hat{V}}_{t}}\right)}_{t=0}^{\infty }$,

$$\begin{array}{cc} E\left[\frac{V_{t+1}\left(\nu \right)}{{\hat{V}}_{t+1}}|F_{t}\right] & =E\left[\int_{L}\frac{V_{t+1}^{\pi }}{{\hat{V}}_{t+1}}d\nu \left(\pi \right)|F_{t}\right]\\ & =\int_{L}E\left[\frac{V_{t+1}^{\pi }}{{\hat{V}}_{t+1}}|F_{t}\right]d\nu \left(\pi \right)\\ & \leq \int_{L}\frac{V_{t}^{\pi }}{{\hat{V}}_{t}}d\nu \left(\pi \right)=\frac{V_{t}\left(\nu \right)}{{\hat{V}}_{t}}. \end{array}$$

□

Here we establish the supermartingale property used in the previous proof.

Lemma 3.13 Let $\hat{\pi }$ be given by (18). Then for every $\pi :\Delta^{d}\to {\bar{\Delta }}^{d}$ and every $x\in \Delta^{d}$,

$$\int_{\Delta^{d}}\frac{\left\langle \pi \left(x\right),\frac{y}{x}\right\rangle }{\left\langle \hat{\pi }\left(x\right),\frac{y}{x}\right\rangle }\varrho \left(x,dy\right)\leq 1.$$

We proceed as in the proof of Becherer (2001, proposition 4.3). Fix π and α∈(0,1) and define $\pi^{\alpha }=\alpha \pi +\left(1-\alpha \right)\hat{\pi }$. Then by the (long only) log‐optimality of $\hat{\pi }$ we have for every $x\in \Delta^{d}$

$$\begin{array}{cc} 0 & \leq \int_{\Delta^{d}}\left(\log〈\hat{\pi }\left(x\right),\frac{y}{x}〉-\log〈\pi^{\alpha }\left(x\right),\frac{y}{x}〉\right)\varrho \left(x,dy\right)=\int_{\Delta^{d}}\left(\int_{〈\pi^{\alpha }\left(x\right),\frac{y}{x}〉}^{〈\hat{\pi }\left(x\right),\frac{y}{x}〉}\frac{1}{z}dz\right)\varrho \left(x,dy\right)\\ & \leq \int_{\Delta^{d}}\frac{\left\langle \hat{\pi }\left(x\right),\frac{y}{x}\right\rangle -\left\langle \pi^{\alpha }\left(x\right),\frac{y}{x}\right\rangle }{\left\langle \pi^{\alpha }\left(x\right),\frac{y}{x}\right\rangle }\varrho \left(x,dy\right)=\int_{\Delta^{d}}\frac{\left\langle \alpha \left(\hat{\pi }\left(x\right)-\pi \left(x\right)\right),\frac{y}{x}\right\rangle }{\left\langle \pi^{\alpha }\left(x\right),\frac{y}{x}\right\rangle }\varrho \left(x,dy\right). \end{array}$$

Hence,

$$\int_{\Delta^{d}}\frac{\left\langle \pi \left(x\right),\frac{y}{x}\right\rangle }{\left\langle \pi^{\alpha }\left(x\right),\frac{y}{x}\right\rangle }\varrho \left(x,dy\right)\leq \int_{\Delta^{d}}\frac{\left\langle \hat{\pi }\left(x\right),\frac{y}{x}\right\rangle }{\left\langle \pi^{\alpha }\left(x\right),\frac{y}{x}\right\rangle }\varrho \left(x,dy\right)\leq \int_{\Delta^{d}}\frac{\left\langle \hat{\pi }\left(x\right),\frac{y}{x}\right\rangle }{\left\langle \left(1-\alpha \right)\hat{\pi }\left(x\right),\frac{y}{x}\right\rangle }\varrho \left(x,dy\right),$$

where the last equality follows from $\pi^{\alpha }\geq \left(1-\alpha \right)\hat{\pi }$. By Fatou's lemma, we therefore have

$$\begin{array}{cc} \int_{\Delta^{d}}\frac{\left\langle \pi \left(x\right),\frac{y}{x}\right\rangle }{\left\langle \hat{\pi }\left(x\right),\frac{y}{x}\right\rangle }\varrho \left(x,dy\right) & =\int_{\Delta^{d}}\lim_{\alpha \to 0}\frac{\left\langle \pi \left(x\right),\frac{y}{x}\right\rangle }{\left\langle \pi^{\alpha }\left(x\right),\frac{y}{x}\right\rangle }\varrho \left(x,dy\right)\leq \lim_{\alpha \to 0}\int_{\Delta^{d}}\frac{\left\langle \pi \left(x\right),\frac{y}{x}\right\rangle }{\left\langle \pi^{\alpha }\left(x\right),\frac{y}{x}\right\rangle }\varrho \left(x,dy\right)\\ & \leq \lim_{\alpha \to 0}\frac{1}{1-\alpha }\int_{\Delta^{d}}\frac{\left\langle \hat{\pi }\left(x\right),\frac{y}{x}\right\rangle }{\left\langle \hat{\pi }\left(x\right),\frac{y}{x}\right\rangle }\varrho \left(x,dy\right)=1. \end{array}$$

□

## 4 THE CONTINUOUS TIME CASE WITH FUNCTIONALLY GENERATED PORTFOLIOS

This section is dedicated to a similar analysis in continuous time and with *functionally generated* portfolio maps (Fernholz, 2002, chapter 3). Using the pathwise Itô calculus developed by Föllmer (1981), we can define the corresponding wealth processes in a pathwise manner for any continuous market path admitting a quadratic variation process. This allows us to define the best retrospectively chosen portfolio which is not well‐defined in general (and in particular for the Lipschitz portfolio maps).

### 4.1 Functionally generated portfolios

We consider the following set of concave functions. For some fixed M>0 and 0≤α≤1, we define

GM,α=G∈C2,α(Δ¯d), concave  such  that ∥G∥C2,α≤M and G≥1M,

where $C^{2,\alpha }\left({\bar{\Delta }}^{d}\right)$ denotes the Hölder space of 2‐times continuously differentiable functions from ${\bar{\Delta }}^{d}\to R$ whose derivatives are α‐Hölder continuous. That is,

$$C^{2,\alpha }\left({\bar{\Delta }}^{d}\right)=\{G\in C^{2}\left({\bar{\Delta }}^{d}\right){\;|\;∥G∥}_{C^{2,\alpha }}<\infty \},$$

where

$${∥G∥}_{C^{2,\alpha }}=\max_{|k|\leq 2}{∥D^{k}G∥}_{\infty }+\max_{|k|=2}\underset{x\neq y}{\sup}\frac{|D^{k}G\left(x\right)-D^{k}G\left(y\right)|}{{∥x-y∥}^{\alpha }}$$

with k denoting a multi‐index in $N^{2}$. For α=0, the second term in this norm is left away. Note that *G* is only defined on the simplex $\Delta^{d}$. In order that the partial derivatives are well‐defined, we assume that each *G* is extended to an open neighborhood of $\Delta^{d}$ such that $G\left(x\right)=G\left(x^{\prime }\right)$, where $x^{\prime }$ is the orthogonal projection of *x* onto $\Delta^{d}$. The choice of the extension is irrelevant.

Here is an analytical lemma whose proof is given in the appendix.

Lemma 4.1 For any M,α>0, the set $G^{M,\alpha }$ is compact with respect to ${∥\cdot ∥}_{C^{2,0}}$.

To the set of generating functions $G^{M,\alpha }$, we associate now the set of functionally generated portfolios ${\mathrm{FG}}^{M,\alpha }$ in the spirit of Fernholz (2002) defined by

(40) $$\begin{array}{ccc} {\mathrm{FG}}^{M,\alpha } & = & \{\pi^{G}:\Delta^{d}\to {\bar{\Delta }}^{d},\\ & & x\mapsto {\left(\pi^{G}\left(x\right)\right)}^{i}=x^{i}\left(\frac{D^{i}G\left(x\right)}{G(x)}+1-\sum_{j=1}^{d}\frac{D^{j}G\left(x\right)}{G(x)}x^{j}\right),\;i=1,\ldots d,\;|\;G\in G^{M,\alpha }\}.\; \end{array}$$

By the concavity of *G*, $\pi^{G}$ takes values in ${\bar{\Delta }}^{d}$, that is, it is long‐only (see, e.g., Fernholz & Karatzas, 2009, remark 11.1). The corresponding wealth processes are denoted by $V^{\pi^{G}}$ or $V^{G}$.

For these portfolios, it is possible to obtain a pathwise expression for $V^{\pi^{G}}$. We refer the reader to Schied et al. (2016) for extensions of this pathwise approach to time‐dependent and path‐dependent generating functions. There, this is achieved by applying the functional Itô calculus developed by Dupire (2009) and Cont and Fournié (2010, 2013), which generalizes Föllmer's Itô calculus to path‐dependent functionals. In this paper, we only consider functionally generated portfolio maps as defined in (40).

We adopt the notation of Schied et al. (2016) and fix a refining sequence of partitions ${\left(T_{n}\right)}_{n=1}^{\infty }$ of [0, ∞), that is, $T_{n}=\left\{t_{0},t_{1},\ldots \right\}$ is such that $0=t_{0}^{n}<t_{1}^{n}<\cdots$ and $t_{k}^{n}\to \infty$ as k→∞, and $T_{1}\subset T_{2}\subset \cdots$. Moreover, the mesh of $T_{n}$ tends to zero on each compact interval as n→∞. Furthermore, we denote the successor of $t\in T_{n}$ by $t^{\prime }$. That is, $t^{\prime }=\min\left\{u\in T_{n}\;|,\;u>t\right\}$. Throughout this section, the market weights are described by a *d*‐dimensional continuous path $\mu ={\left(\mu_{t}\right)}_{t\geq 0}$ with values in $\Delta^{d}$. Here and henceforth we let $S_{d}^{+}$ be the set of d×d positive definite matrices.

Assumption 4.2 The path ${\left(\mu_{t}\right)}_{t\geq 0}$ admits a continuous $S_{d}^{+}$‐valued quadratic variation [μ] along $\left(T_{n}\right)$ in the sense of Föllmer (1981), that is, for any 1≤i,j≤d and all t≥0 the sequence

$$\sum_{s\in T_{n},s\leq t}\left(\mu_{s^{\prime }}^{i}-\mu_{s}^{i}\right)\left(\mu_{s^{\prime }}^{j}-\mu_{s}^{j}\right)$$

converges to a finite limit, as n→∞, denoted ${\left[\mu^{i},\mu^{j}\right]}_{t}$, such that $t\mapsto {\left[\mu^{i},\mu^{j}\right]}_{t}$ is continuous.

The dynamics of the relative wealth process $V^{\pi^{G}}$ built by investing according to $\pi^{G}\in {\mathrm{FG}}^{M,\alpha }$ are given in this continuous‐time case by

(41) $$\begin{array}{c} \frac{dV_{t}^{\pi^{G}}}{V_{t}^{\pi^{G}}}=\sum_{i=1}^{d}{\left(\pi^{G}\left(\mu_{t}\right)\right)}^{i}\frac{d\mu_{t}^{i}}{\mu_{t}^{i}}=\sum_{i=1}^{d}\frac{D^{i}G\left(\mu_{t}\right)}{G(\mu_{t})}d\mu_{t}^{i},\;V_{0}^{\pi }=1, \end{array}$$

(compare (11) in the discrete time case), where the right‐hand side has to be understood as Föllmer's pathwise integral (cf.  equation (6) in Schied et al., 2016). Note that the second equality holds by the definition of $\pi^{G}$ and the fact that $\sum_{i=1}^{d}d\mu_{t}^{i}=0$.

Using (41) and Föllmer's Itô calculus, we have the following pathwise version of Fernholz's (2002) master equation (also see Schied et al., 2016, theorem 2.9).

Corollary 4.3 Let $G\in C^{2}\left({\bar{\Delta }}^{d}\right)$ and $\pi^{G}$ be defined as in (40). Let ${\left(\mu_{t}\right)}_{t\geq 0}$ be a continuous path satisfying Assumption 4.2. Then $V^{\pi^{G}}$ satisfies

(42) $$\begin{array}{c} V_{T}^{\pi^{G}}\equiv V_{T}^{G}=\frac{G(\mu_{T})}{G(\mu_{0})}e^{g([0,T])},\;0\leq T<\infty , \end{array}$$

where $g\left(dt\right)=-\frac{1}{2G(\mu_{t})}\sum_{i,j}D^{ij}G\left(\mu_{t}\right)d{\left[\mu^{i},\mu^{j}\right]}_{t}$.

### 4.2 Definitions of the portfolios

We again consider (i) the best retrospectively chosen portfolio, (ii) the universal portfolio, and (iii) the log‐optimal portfolio. To define the log‐optimal portfolio, we will restrict to a specific stochastic model introduced in Section 4.2.3. In Section 4.2.4, we derive the asymptotic growth rate for this model class under an additional ergodicity assumption.

#### 4.2.1 The best retrospectively chosen portfolio

We consider the set of functionally generated portfolios ${\mathrm{FG}}^{M,\alpha }$ and a given continuous path ${\left(\mu_{t}\right)}_{t\geq 0}$ satisfying Assumption 4.2. For M,α>0 fixed, we define

(43) $$\begin{array}{c} V_{T}^{*,M,\alpha }=\underset{\pi^{G}\in {\mathrm{FG}}^{M,\alpha }}{\sup}V_{T}^{\pi^{G}}=\underset{G\in G^{M,\alpha }}{\sup}V_{T}^{G}. \end{array}$$

We first prove that an optimizer exists by establishing the following continuity property whose proof can be found in the appendix.

Lemma 4.4 Let T,M,α>0 be fixed and ${\left(\mu_{t}\right)}_{t\geq 0}$ be a continuous path satisfying Assumption 4.2. Consider the function $G\mapsto V_{T}^{G}$ where $V_{T}^{G}$ is given by (42). Then $G\mapsto V_{T}^{G}$ is continuous from $\left(G^{M,\alpha },∥\cdot {∥}_{C^{2,0}}\right)$ to R.

Proposition 4.5 Let *T* be fixed and ${\left(\mu_{t}\right)}_{t\geq 0}$ be a continuous path satisfying Assumption 4.2. Let $V_{T}^{*,M,\alpha }$ be defined by (43). Then there exists an optimizer $G_{T}^{*}\in G^{M,\alpha }$and in turn a portfolio $\pi_{T}^{*}$ generated by $G_{T}^{*}$ such that

$$V_{T}^{*,M,\alpha }=V_{T}^{\pi_{T}^{*}}=V_{T}^{G_{T}^{*}}.$$

This is simply a consequence of continuity as proved in Lemma 4.4 and compactness of $\left(G^{M,\alpha },∥\cdot {∥}_{C^{2,0}}\right)$ as shown in Lemma 4.1. □

#### 4.2.2 Universal portfolio

To define the analog of Cover's/Jamshidian's portfolio in the present setting, let *m* be a Borel probability measure on $\left(G^{M,\alpha },∥\cdot {∥}_{C^{2,0}}\right)$. Consider the map

(44) $$\begin{array}{c} F:G^{M,\alpha }\to {\mathrm{FG}}^{M,\alpha };\;G\mapsto F\left(G\right)=\pi^{G}, \end{array}$$

where $\pi^{G}$ is given by (40). Define now on $\left({\mathrm{FG}}^{M,\alpha },∥\cdot {∥}_{\infty }\right)$ a Borel probability measure ν via the pushforward $\nu =F_{*}m$. As in Definition 2.4, we then define the corresponding universal portfolio via

(45) $$\begin{array}{c} \pi_{T}^{\nu }=\frac{\int_{{\mathrm{FG}}^{M,\alpha }}\pi^{G}\left(\mu_{T}\right)V_{T}^{\pi^{G}}d\nu \left(\pi^{G}\right)}{\int_{{\mathrm{FG}}^{M,\alpha }}V_{T}^{\pi^{G}}d\nu \left(\pi^{G}\right)}. \end{array}$$

Analogous to (14), the value of the universal portfolio is given by

(46) $$\begin{array}{c} V_{T}^{M,\alpha }\left(\nu \right):=V_{T}^{\pi^{\nu }}=\int_{{\mathrm{FG}}^{M,\alpha }}V_{T}^{\pi^{G}}d\nu \left(\pi^{G}\right)=\int_{G^{M,\alpha }}V_{T}^{G}dm\left(G\right). \end{array}$$

Remark 4.6 More precisely, we need to verify that the universal portfolio still allows for pathwise integration and that the value of the portfolio (as a pathwise integral) is given by the right‐hand side of (46). These claims can be easily checked using the definitions and results in Schied et al. (2016), so we omit the details.

#### 4.2.3 Functionally generated log‐optimal portfolios

By definition, the log‐optimal portfolios requires a stochastic model for the market weights. We suppose that $\mu ={\left(\mu_{t}^{1},\ldots ,\mu_{t}^{d}\right)}_{t\geq 0}$ follows a *time‐homogeneous Markovian Itô diffusion*, defined on $\left(\Omega ,F,{\left(F_{t}\right)}_{t\geq 0},P\right)$ with values in $\Delta^{d}$, given by

(47) $$\begin{array}{c} \mu_{t}=\mu_{0}+\int_{0}^{t}c\left(\mu_{s}\right)\lambda \left(\mu_{s}\right)ds+\int_{0}^{t}\sqrt{c(\mu_{s})}dW_{s},\;\mu_{0}\in \Delta^{d}, \end{array}$$

where $\sqrt{\cdot }$ denotes the matrix square root, *W* is a *d*‐dimensional Brownian motion, λ is a Borel measurable function from $\Delta^{d}\to R^{d}$, and *c* is a Borel measurable function from $\Delta^{d}\to S_{+}^{d}$, satisfying

(48) $$\begin{array}{cc} & \int_{0}^{T}\lambda^{⊤}\left(\mu_{t}\right)c\left(\mu_{t}\right)\lambda \left(\mu_{t}\right)dt<\infty ,\;\forall T\in \left[0,\infty \right), \end{array}$$

(49) $$\begin{array}{cc} & c\left(x\right)1=0,\;\sum_{i,j}c^{ij}\left(x\right)\lambda {\left(x\right)}^{j}=0,\;\forall x\in \Delta^{d}. \end{array}$$

The requirements in (49) are necessary to guarantee that the process μ lies in $\Delta^{d}$. Note that ${\left(\mu_{t}\right)}_{t\geq 0}$ given by (47) satisfies the so‐called *structure condition* (see Schweizer, 1995) (because of (48) and the fact that the drift part is of form $\int_{0}^{t}c\left(\mu_{s}\right)\lambda \left(\mu_{s}\right)ds$). This structural condition characterizes the condition of “no unbounded profit with bounded risk” (NUPBR) in the case of continuous semimartingales (see, e.g., Hulley & Schweizer, 2010).

In this setting, the proportions of current (relative) wealth invested in each of the assets are described by processes π in the following set:

(50) $$\begin{array}{c} \Pi =\{\pi \;|\;H^{d}\text{-valued,}\;\text{predictable},R\text{-integrable}\}, \end{array}$$

where the process *R* is defined componentwise by $R_{t}^{i}=\int_{0}^{t}\frac{d\mu_{s}^{i}}{\mu_{s}^{i}}$. Here, $H^{d}$ denotes the hyperplane corresponding to portfolio weights that are *not* necessarily long‐only, that is, $H^{d}=\left\{x\in R^{d}|\sum_{j=1}^{d}x^{j}=1\right\}$. Note that the set ${\mathrm{FG}}^{M,\alpha }$ is clearly a subset of long‐only strategies in Π. The relative wealth process $V^{\pi }$ satisfies

(51) $$\begin{array}{c} \frac{dV_{t}^{\pi }}{V_{t}^{\pi }}=\sum_{i=1}^{d}\pi_{t}^{i}\frac{d\mu_{t}^{i}}{\mu_{t}^{i}},\;V_{0}^{\pi }=1. \end{array}$$

In contrast to Section 4.1, this is a usual stochastic integral because we are dealing with general integrands π. Note that we can also write

(52) $$\begin{array}{cc} V_{T}^{\pi } & =E{\left(\left(\pi •R\right)\right)}_{T}=\exp\left(\int_{0}^{T}{\left(\frac{\pi }{\mu_{t}}\right)}^{⊤}d\mu_{t}-\frac{1}{2}\int_{0}^{T}{\left(\frac{\pi }{\mu_{t}}\right)}^{⊤}c\left(\mu_{t}\right)\frac{\pi }{\mu_{t}}dt\right)\\ & =\exp\left(\int_{0}^{T}\sum_{i=1}^{d}\pi^{i}\frac{d\mu_{t}^{i}}{\mu_{t}^{i}}-\frac{1}{2}\int_{0}^{T}\sum_{i,j}\frac{\pi^{i}}{\mu_{t}^{i}}\frac{\pi^{j}}{\mu_{t}^{j}}c^{ij}\left(\mu_{t}\right)dt\right), \end{array}$$

where, for two vectors $p,q\in R^{d}$, p/q always denotes the componentwise quotient $\left(\frac{p^{1}}{q^{1}},\ldots ,\frac{p^{d}}{q^{d}}\right)$.

Next we consider the log‐optimal portfolio defined by (15) (but in continuous time now). As in Fernholz and Karatzas (2010b, section 3.1), we derive the ratio of two wealth processes $V^{\pi }$ and $V^{\theta }$ for π,θ∈Π. Using (51) (for the processes π and θ) and Itô's lemma, this ratio is given by

$$\begin{array}{cc} d\left(\frac{V_{t}^{\pi }}{V_{t}^{\theta }}\right) & =\frac{V_{t}^{\pi }}{V_{t}^{\theta }}{\left(\frac{\pi_{t}}{\mu_{t}}-\frac{\theta_{t}}{\mu_{t}}\right)}^{⊤}\left(d\mu_{t}-c\left(\mu_{t}\right)\frac{\theta_{t}}{\mu_{t}}dt\right)\\ & =\frac{V_{t}^{\pi }}{V_{t}^{\theta }}{\left(\frac{\pi_{t}}{\mu_{t}}-\frac{\theta_{t}}{\mu_{t}}\right)}^{⊤}\left(\sqrt{c(\mu_{t})}dW_{t}+c\left(\mu_{t}\right)\left(\lambda \left(\mu_{t}\right)-\frac{\theta_{t}}{\mu_{t}}\right)dt\right). \end{array}$$

The finite variation part of the expression vanishes *for every* π∈Π if we choose θ∈Π such that

(53) $$\begin{array}{c} c\left(\mu_{t}\right)\left(\frac{\theta_{t}}{\mu_{t}}-\lambda \left(\mu_{t}\right)\right)=0,\;P\text{-a.s.}\;\text{for}\;\text{all}\;t\geq 0. \end{array}$$

By passing from the scaled relative weights θ/μ to ordinary portfolio weights via Fernholz and Karatzas (2010b, equation (5)), the generic solution of (53), which we denote by $\hat{\pi }$,^8^ is given by

(54) $$\begin{array}{c} {\hat{\pi }}_{t}^{i}=\mu_{t}^{i}\left(\lambda^{i}\left(\mu_{t}\right)+1-\sum_{j=1}^{d}\mu_{t}^{j}\lambda^{j}\left(\mu_{t}\right)\right). \end{array}$$

Let $\hat{V}$ be the associated wealth process. From (53), the ratio $V_{t}^{\pi }/{\hat{V}}_{t}$ is, for any π∈Π, a nonnegative local martingale and therefore a supermartingale. Hence ${\hat{V}}_{t}$ yields the relative wealth process corresponding to the log‐optimal portfolio (see, e.g., Fernholz & Karatzas, 2010b; Karatzas & Kardaras, 2007). Indeed, by the supermartingale property and Jensen's inequality

$$E\left[\log\left(V_{T}^{\pi }\right)-\log\left({\hat{V}}_{T}\right)\right]=E\left[\log\left(\frac{V_{T}^{\pi }}{{\hat{V}}_{T}}\right)\right]\leq \log\left(E\left[\frac{V_{T}^{\pi }}{{\hat{V}}_{T}}\right]\right)\leq 0.$$

Thus $E\left[\log\left(V_{T}^{\pi }\right)\right]\leq E\left[\log\left({\hat{V}}_{T}\right)\right]$ for all π∈Π.

By (52), the expected value of the log‐optimal portfolio is given by

$$\underset{\pi \in \Pi }{\sup}E\left[\log V_{T}^{\pi }\right]=\frac{1}{2}E\left[\int_{0}^{T}\lambda^{⊤}\left(\mu_{t}\right)c\left(\mu_{t}\right)\lambda \left(\mu_{t}\right)dt\right].$$

So far we have optimized over *all* strategies in Π. In the sequel, we shall mainly consider suprema taken over smaller sets, in particular over ${\mathrm{FG}}^{M,\alpha }$. Note that in this case the optimizer will still be a function of the market weights due to the Markov property of ${\left(\mu_{t}\right)}_{t\geq 0}$.

In this context, let us also answer the question of when the log‐optimal portfolio is functionally generated. This is needed to relate its asymptotic growth rate to the one of the best retrospectively chosen portfolio and the universal portfolio.

Proposition 4.7 Let ${\left(\mu_{t}\right)}_{t\geq 0}$ be of the form (47). Then the log‐optimal portfolio is generated by a differentiable function *G*, that is,

$$\begin{array}{c} {\hat{\pi }}_{t}^{i}=\mu_{t}^{i}\left(\frac{D^{i}G\left(\mu_{t}\right)}{G(\mu_{t})}+1-\sum_{j=1}^{d}\mu_{t}^{j}\frac{D^{j}G\left(\mu_{t}\right)}{G(\mu_{t})}\right),\;i=1,\ldots ,d, \end{array}$$

if the drift characteristic λ satisfies

$$\lambda \left(x\right)=\nabla \log G\left(x\right)=\frac{\nabla G(x)}{G(x)},\;x\in \Delta^{d}.$$

The assertion follows from expression (54). □

#### 4.2.4 Asymptotic growth rates for an ergodic market weights process

Assumption 4.8 The process μ as given in (47) is an ergodic process with stationary measure ϱ on $\Delta^{d}$.

With this assumption we derive an expression of the asymptotic growth rate $\lim_{T\to \infty }\frac{1}{T}\log V_{T}^{\pi }$. For the precise notion of ergodicity in continuous time, we refer to Eberle (2016, section 2.2., theorem 2.4, and section 2.2.3). Assumption 4.8 is essentially satisfied under a mean reversion condition. Examples include polynomial models for the market weights staying in the interior of the simplex (see Cuchiero, 2019, theorem 5.1) with the subclass of volatility stabilized models (Fernholz & Karatzas, 2005).

In the following theorem, we consider portfolio maps which are not necessarily long‐only, but can take values in the hyperplane $H^{d}$.

Theorem 4.9 Under Assumption 4.8 the following statements hold true:

(i) Let $\pi :\Delta^{d}\to H^{d}$ be any (ϱ‐measurable) portfolio map such that
  (55) $$\begin{array}{c} \int_{\Delta^{d}}\left|{\left(\frac{\pi (x)}{x}\right)}^{⊤}c\left(x\right)\lambda \left(x\right)\right|\varrho \left(dx\right)<\infty ,\\ Q^{\pi }:=\int_{\Delta^{d}}{\left(\frac{\pi (x)}{x}\right)}^{⊤}c\left(x\right)\left(\frac{\pi (x)}{x}\right)\varrho \left(dx\right)<\infty . \end{array}$$
  We then have, for ϱ‐a.e. starting value μ_0_, that
  $$\begin{array}{cc} \lim_{T\to \infty }\frac{1}{T}\log\left(V_{T}^{\pi }\right) & =L^{\pi }:=\int_{\Delta^{d}}{\left(\frac{\pi (x)}{x}\right)}^{⊤}c\left(x\right)\lambda \left(x\right)\varrho \left(dx\right)\\ & -\frac{1}{2}\int_{\Delta^{d}}{\left(\frac{\pi (x)}{x}\right)}^{⊤}c\left(x\right)\left(\frac{\pi (x)}{x}\right)\varrho \left(dx\right),\;P-a.s. \end{array}$$
(ii) Assume that $L:=\frac{1}{2}\int_{\Delta^{d}}\lambda^{⊤}\left(x\right)c\left(x\right)\lambda \left(x\right)\varrho \left(dx\right)<\infty$. Then, for ϱ‐a.e. starting value μ_0_, it holds that
  $$\lim_{T\to \infty }\frac{1}{T}\log{\hat{V}}_{T}=L,\;P-a.s.$$

The proof of Theorem 4.9 relies on the following lemma which is stated and proved in Fernholz (2002, lemma 1.3.2).

Lemma 4.10 Let *M* be a continuous local martingale such that

(56) $$\begin{array}{c} \lim_{T\to \infty }\frac{1}{T^{2}}{\left\langle M,M\right\rangle }_{T}\log\log T=0,\;P-a.s. \end{array}$$

Then $\lim_{T\to \infty }\frac{1}{T}M_{T}=0$, P‐a.s.

Proof of Theorem 4.9 Let us start by proving statement (i). By (51), $\log V_{T}^{\pi }$ reads as

(57) $$\begin{array}{ccc} \log V_{T}^{\pi } & = & \int_{0}^{T}{\left(\frac{\pi (\mu_{t})}{\mu_{t}}\right)}^{⊤}c\left(\mu_{t}\right)\lambda \left(\mu_{t}\right)dt-\frac{1}{2}\int_{0}^{T}{\left(\frac{\pi (\mu_{t})}{\mu_{t}}\right)}^{⊤}c\left(\mu_{t}\right)\frac{\pi (\mu_{t})}{\mu_{t}}dt\\ & & +\int_{0}^{T}{\left(\frac{\pi (\mu_{t})}{\mu_{t}}\right)}^{⊤}\sqrt{c(\mu_{t})}dW_{t}. \end{array}$$

The local martingale part

$$M_{T}^{\pi }:=\int_{0}^{T}{\left(\frac{\pi (\mu_{t})}{\mu_{t}}\right)}^{⊤}\sqrt{c(\mu_{t})}dW_{t}$$

satisfies Condition (56) of Lemma 4.10. Indeed, by the ergodic theorem in continuous time (see, e.g., Eberle, 2016, theorem 2.4 and section 2.2.3) and (55) we have

$$\frac{1}{T}{\left\langle M^{\pi },M^{\pi }\right\rangle }_{T}=\frac{1}{T}\int_{0}^{T}{\left(\frac{\pi (\mu_{t})}{\mu_{t}}\right)}^{⊤}c\left(\mu_{t}\right)\frac{\pi (\mu_{t})}{\mu_{t}}dt\overset{T\to \infty }{\to }Q^{\pi }<\infty ,\;P-a.s.$$

Multiplying the left‐hand side with (loglogT)/T, therefore yields Condition (56) and

$$\frac{1}{T}M_{T}^{\pi }=\frac{1}{T}\int_{0}^{T}{\left(\frac{\pi (\mu_{t})}{\mu_{t}}\right)}^{⊤}\sqrt{c(\mu_{t})}dW_{t}\to 0,\;P-a.s.$$

Hence, evoking again the ergodic theorem yields

$$\begin{array}{cc} \lim_{T\to \infty }\frac{1}{T}\log V_{T}^{\pi } & =\lim_{T\to \infty }\frac{1}{T}\left(\int_{0}^{T}{\left(\frac{\pi (\mu_{t})}{\mu_{t}}\right)}^{⊤}c\left(\mu_{t}\right)\lambda \left(\mu_{t}\right)dt-\frac{1}{2}\int_{0}^{T}{\left(\frac{\pi (\mu_{t})}{\mu_{t}}\right)}^{⊤}c\left(\mu_{t}\right)\frac{\pi (\mu_{t})}{\mu_{t}}dt\right)\\ & =\int_{\Delta^{d}}{\left(\frac{\pi (x)}{x}\right)}^{⊤}c\left(x\right)\lambda \left(x\right)\varrho \left(dx\right)-\frac{1}{2}\int_{\Delta^{d}}{\left(\frac{\pi (x)}{x}\right)}^{⊤}c\left(x\right)\left(\frac{\pi (x)}{x}\right)\varrho \left(dx\right), \end{array}$$

P‐a.s. (and also in $L^{1}\left(\Omega ,F,P\right)$) and thus assertion (i).

Concerning statement (ii), note from (53) that the scaled relative weights corresponding to the log‐optimal portfolio satisfy

$$c\left(x\right)\left(\frac{\hat{\pi }\left(x\right)}{x}-\lambda \left(x\right)\right)=0.$$

Thus, by (57) and (51), $\log{\hat{V}}_{T}$ simplifies to

$$\log{\hat{V}}_{T}=\frac{1}{2}\int_{0}^{T}\lambda^{⊤}\left(\mu_{t}\right)c\left(\mu_{t}\right)\lambda \left(\mu_{t}\right)dt+\int_{0}^{T}\lambda^{⊤}\left(\mu_{t}\right)\sqrt{c(\mu_{t})}dW_{t}.$$

In this case, we have

$$\frac{1}{T}{\left\langle M^{\hat{\pi }},M^{\hat{\pi }}\right\rangle }_{T}=\frac{1}{T}\int_{0}^{T}\lambda^{⊤}\left(\mu_{t}\right)c\left(\mu_{t}\right)\lambda \left(\mu_{t}\right)dt\overset{T\to \infty }{\to }2L,\;P-a.s.,$$

which yields by the same argument as above

$$\frac{1}{T}M_{T}^{\hat{\pi }}=\frac{1}{T}\int_{0}^{T}\lambda^{⊤}\left(\mu_{t}\right)\sqrt{c(\mu_{t})}dW_{t}\to 0,\;P-a.s.$$

and in turn

$$\lim_{T\to \infty }\frac{1}{T}\log{\hat{V}}_{T}=\lim_{T\to \infty }\frac{1}{2T}\int_{0}^{T}\lambda^{⊤}\left(\mu_{t}\right)c\left(\mu_{t}\right)\lambda \left(\mu_{t}\right)dt=L,\;P-a.s.$$

□

### 4.3 Asymptotically equivalent growth rates

As in discrete time, we will establish asymptotic equality of the growth rates of all three portfolio types introduced in Section 4.2. First, we compare the best retrospectively chosen portfolio with the universal one. For an analogous result in the context of optimal arbitrage, see theorem 4.5 of Kardaras and Robertson (2012).

Theorem 4.11 Let M,α>0 be fixed and let ${\left(\mu_{t}\right)}_{t\geq 0}$ be a continuous path satisfying Assumption 4.2 such that for all i∈{1,…,d}

(58) $$\begin{array}{c} \lim_{T\to \infty }\frac{1}{T}{\left[\mu^{i},\mu^{i}\right]}_{T}<\infty . \end{array}$$

Consider a probability measure *m* on $G^{M,\alpha }$ with full support and set $\nu =F_{*}m$ with *F* defined in (44). Then

$$\lim_{T\to \infty }\frac{1}{T}\left(\log V_{T}^{*,M,\alpha }-\log V_{T}^{M,\alpha }\left(\nu \right)\right)=0,$$

where $V^{*,M,\alpha }$ and $V^{M,\alpha }\left(\nu \right)$ are defined in (43) and (46), respectively.

The inequality “≥” is obvious. For the converse inequality, we proceed similarly as in the previous section (using only generating functions). As *m* has full support and $G^{M,\alpha }$ is compact, we have that, for η>0 there exists some δ>0, such that every η‐neighborhood of a point $G\in G^{M,\alpha }$ has *m*‐measure bigger than δ.

Let T≥1 and denote by $G_{T}^{*}$ the optimizer as of Proposition 4.5. Consider now a generating function *G* such $∥G-G_{T}^{*}{∥}_{C^{2,0}}\leq \eta$. Then it follows from (A9) that

(59) $$\begin{array}{ccc} \frac{1}{T}\left(\log\left(V_{T}^{G}\right)-\log\left(V_{T}^{G_{T}^{*}}\right)\right) & \geq & \frac{1}{T}\left(-2M\eta -\left(\frac{M}{2}d^{2}\eta +\frac{M^{3}}{2}d^{2}\eta \right)\max_{i}{\left[\mu^{i},\mu^{i}\right]}_{T}\right)\\ & = & :-K_{T}. \end{array}$$

Fix ε>0 and note that by assumption (58) and continuity of $T\mapsto \frac{1}{T}{\left[u^{i},u^{i}\right]}_{T}$ on [1, ∞), $\sup_{T\in [1,\infty )}\frac{1}{T}{\left[\mu^{i},\mu^{i}\right]}_{T}$ can be bounded by some constant. Therefore, we can choose η sufficiently small such that $K_{T}\leq \varepsilon$ for all T≥1.

Denote by $B=B_{\eta }\left(G_{T}^{*}\right)$ the ${∥\cdot ∥}_{C^{2,0}}$‐ball with radius η in $G^{M,\alpha }$ which has *m*‐measure at least δ>0, where δ only depends on η. We then may estimate using Jensen's inequality and (59)

$$\begin{array}{cc} {\left(\frac{V_{T}^{M,\alpha }\left(\nu \right)}{V_{T}^{G_{T}^{*}}}\right)}^{\frac{1}{T}} & ={\left(\frac{\int_{G^{M,\alpha }}V_{T}^{G}m\left(dG\right)}{V_{T}^{G_{T}^{*}}}\right)}^{\frac{1}{T}}\geq {\left(\frac{\int_{B_{\eta }\left(G_{T}^{*}\right)}V_{T}^{G}m\left(dG\right)}{V_{T}^{G_{T}^{*}}}\right)}^{\frac{1}{T}}\\ & \geq \delta^{\frac{1}{T}-1}\frac{\int_{B_{\eta }\left(G_{T}^{*}\right)}{\left(V_{T}^{G}\right)}^{\frac{1}{T}}m\left(dG\right)}{{\left(V_{T}^{G_{T}^{*}}\right)}^{\frac{1}{T}}}\geq \delta^{\frac{1}{T}}e^{-K_{T}}\geq \delta^{\frac{1}{T}}e^{-\varepsilon }. \end{array}$$

Letting T→∞ for any given ε (which determines η and in turn δ) yields the assertion. □

To compare the asymptotic performance with that of the log‐optimal portfolio, we optimize over portfolio maps in ${\mathrm{FG}}^{M,\alpha }$ and suppose henceforth that ${\left(\mu_{t}\right)}_{t\geq 0}$ is of the form (47). Under Assumption 4.8 and from Theorem 4.9 define

(60) $$\begin{array}{ccc} {\hat{\pi }}^{M,\alpha } & := & \underset{\pi^{G}\in {\mathrm{FG}}^{M,\alpha }}{arg max}\left(\int_{\Delta^{d}}{\left(\frac{\pi^{G}\left(x\right)}{x}\right)}^{⊤}c\left(x\right)\lambda \left(x\right)\varrho \left(dx\right)\right.\\ & & \left.-\frac{1}{2}\int_{\Delta^{d}}{\left(\frac{\pi^{G}\left(x\right)}{x}\right)}^{⊤}c\left(x\right)\left(\frac{\pi^{G}\left(x\right)}{x}\right)\varrho \left(dx\right)\right) \end{array}$$

and the corresponding wealth process ${\hat{V}}^{M,\alpha }$ by ${\hat{V}}^{M,\alpha }=V^{{\hat{\pi }}^{M,\alpha }}$, whenever ${\hat{\pi }}^{M,\alpha }$ is well‐defined. As

$$\underset{\pi^{G}\in {\mathrm{FG}}^{M,\alpha }}{\sup}E\left[\log\left(V_{T}^{\pi^{G}}\right)\right]$$

yields ${\hat{\pi }}^{M,\alpha }$ as optimizer for all T>0, ${\hat{V}}^{M,\alpha }$ corresponds to the log‐optimal portfolio among functionally generated portfolios with generating function in $G^{M,\alpha }$.

Theorem 4.12 Let M,α>0 be fixed and let ${\left(\mu_{t}\right)}_{t\geq 0}$ be a stochastic process of the form (47) satisfying Assumption 4.8. Moreover, suppose that

(61) $$\begin{array}{cc} & \int_{\Delta^{d}}c^{ii}\left(x\right)\varrho \left(dx\right)<\infty ,\;\text{for}\;\text{all}\;i\in \left\{1,\ldots ,d\right\}, \end{array}$$

(62) $$\begin{array}{cc} & \int_{\Delta^{d}}\max_{i\in \{1,\ldots ,d\}}\left|{\left(c\left(x\right)\lambda \left(x\right)\right)}^{i}\right|\varrho \left(dx\right)<\infty . \end{array}$$

Consider a probability measure *m* on $G^{M,\alpha }$ with full support and set $\nu =F_{*}m$ with *F* defined in (44). Then

(63) $$\begin{array}{c} \underset{T\to \infty }{lim inf}\frac{1}{T}\log V_{T}^{*,M,\alpha }=\underset{T\to \infty }{lim inf}\frac{1}{T}\log V_{T}^{M,\alpha }\left(\nu \right)=\lim_{T\to \infty }\frac{1}{T}\log{\hat{V}}_{T}^{M,\alpha },\;P-a.s. \end{array}$$

where ${\hat{V}}_{T}^{M,\alpha }$ denotes the log‐optimal portfolio among ${\mathrm{FG}}^{M,\alpha }$‐maps defined via (60), $V^{*,M,\alpha }$ and $V^{M,\alpha }\left(\nu \right)$ are defined pathwise in (43) and (46), respectively.

We first note that ${\hat{\pi }}^{M,\alpha }$ is well‐defined. Indeed, the map

$$\begin{array}{cc} G & \mapsto \int_{\Delta^{d}}{\left(\frac{\pi^{G}\left(x\right)}{x}\right)}^{⊤}c\left(x\right)\lambda \left(x\right)\varrho \left(dx\right)-\frac{1}{2}\int_{\Delta^{d}}{\left(\frac{\pi^{G}\left(x\right)}{x}\right)}^{⊤}c\left(x\right)\left(\frac{\pi^{G}\left(x\right)}{x}\right)\varrho \left(dx\right)\\ & =\int_{\Delta^{d}}{\left(\frac{\nabla G(x)}{G(x)}\right)}^{⊤}c\left(x\right)\lambda \left(x\right)\varrho \left(dx\right)-\frac{1}{2}\int_{\Delta^{d}}{\left(\frac{\nabla G(x)}{G(x)}\right)}^{⊤}c\left(x\right)\left(\frac{\nabla G(x)}{G(x)}\right)\varrho \left(dx\right) \end{array}$$

is continuous from $\left(G^{M,\alpha },∥\cdot {∥}_{2,0}\right)$ to R. This together with compactness of $G^{M,\alpha }$ with respect to ${∥\cdot ∥}_{2,0}$ imply the well‐definedness of ${\hat{\pi }}^{M,\alpha }$.

Note also that (61) and (62) as well as the conditions on *G* imply the assumptions of the ergodic theorem (Theorem 4.9). Hence, we have for each $\pi^{G}\in {\mathrm{FG}}^{M,\alpha }$ the P‐a.s. limit

$$\lim_{T\to \infty }\frac{1}{T}\log V_{T}^{\pi^{G}}=L^{\pi^{G}}.$$

In particular,

(64) $$\begin{array}{c} \lim_{T\to \infty }\frac{1}{T}\log{\hat{V}}_{T}^{M,\alpha }=\underset{\pi^{G}\in {\mathrm{FG}}^{M,\alpha }}{\sup}L^{\pi^{G}}=:L^{M,\alpha } \end{array}$$

holds P‐a.s.

Due to (61), we can now apply Theorem 4.11 which implies the first equality in (63). Moreover, we have by the definition of $V_{T}^{*,M,\alpha }$ for each fixed *T* the inequality

(65) $$\begin{array}{c} \frac{1}{T}\log\left({\hat{V}}_{T}^{M,\alpha }\right)\leq \frac{1}{T}\log\left(V_{T}^{*,M,\alpha }\right),\;P-a.s. \end{array}$$

Using (64), (65), and Theorem 4.11, we thus have P‐a.s.,

(66) $$\begin{array}{c} L^{M,\alpha }=\lim_{T\to \infty }\frac{1}{T}\log\left({\hat{V}}_{T}^{M,\alpha }\right)\leq \underset{T\to \infty }{lim inf}\frac{1}{T}\log\left(V_{T}^{*,M,\alpha }\right)=\underset{T\to \infty }{lim inf}\frac{1}{T}\log\left(V_{T}^{M,\alpha }\left(\nu \right)\right). \end{array}$$

On the other hand, by the definition of ${\left({\hat{V}}_{t}^{M,\alpha }\right)}_{t\geq 0}$ as log‐optimizer within the class ${\mathrm{FG}}^{M,\alpha }$

(67) $$\begin{array}{c} E\left[\log\left(V_{T}^{M,\alpha }\left(\nu \right)\right)\right]\leq \underset{\pi^{G}\in {\mathrm{FG}}^{M,\alpha }}{\sup}E\left[\log\left(V_{T}^{\pi^{G}}\right)\right]=E\left[\log\left({\hat{V}}_{T}^{M,\alpha }\right)\right] \end{array}$$

holds. Concerning the first inequality, note that the universal portfolio to build the wealth $V_{T}^{M,\alpha }\left(\nu \right)$ is given by (45). By the time‐homogenous Markovianity it is thus sufficient to dominate the left‐hand side of (67) by taking the supremum over elements in ${\mathrm{FG}}^{M,\alpha }$.

Combining now (67), Theorem 4.9 and (66) yields,

$$\begin{array}{cc} E\left[\underset{T\to \infty }{lim inf}\frac{1}{T}\log\left(V_{T}^{M,\alpha }\left(\nu \right)\right)\right] & \leq \underset{T\to \infty }{lim inf}\frac{1}{T}E\left[\log\left(V_{T}^{M,\alpha }\left(\nu \right)\right)\right]\\ & \leq \lim_{T\to \infty }\frac{1}{T}E\left[\log\left({\hat{V}}_{T}^{M,\alpha }\right)\right]\\ & =\lim_{T\to \infty }\frac{1}{T}\log\left({\hat{V}}_{T}^{M,\alpha }\right)\\ & \leq \underset{T\to \infty }{lim inf}\frac{1}{T}\log\left(V_{T}^{M,\alpha }\left(\nu \right)\right),\;P\text{-a.s.}, \end{array}$$

where the first inequality follows from Fatou's lemma. From this, we see that

$$\underset{T\to \infty }{lim inf}\frac{1}{T}\log\left(V_{T}^{M,\alpha }\left(\nu \right)\right)$$

is P‐a.s. constant and equal to $\lim_{T\to \infty }\frac{1}{T}\log\left({\hat{V}}_{T}^{M,\alpha }\right)$. Hence the assertion is proved. □

As in the previous section, we can formulate a result not depending explicitly on the constant *M* on α. Setting $\alpha =\frac{1}{M}$ we choose for M=1,2,3,⋯ a measure $m^{M}$ on $G^{M,\frac{1}{M}}$ with full support. Define $m=\sum_{M=1}^{\infty }2^{-M}m^{M}$ and the process V(ν) by

$$\begin{array}{c} V_{T}\left(\nu \right)=\int_{{⋃}_{M=1}^{\infty }G^{M,\frac{1}{M}}}V_{T}^{G}m\left(dG\right). \end{array}$$

In order to compare the performance with the one of the global log‐optimal portfolio, whenever it is functionally generated, we combine the above results with Proposition 4.7.

Corollary 4.13 Let ${\left(\mu_{t}\right)}_{t\geq 0}$ be a stochastic process of form (47) satisfying Assumption 4.8. Moreover, suppose that λ and *c* satisfy (61) and

(68) $$\begin{array}{cc} & \lambda \left(x\right)=\frac{\nabla \hat{G}\left(x\right)}{\hat{G}\left(x\right)}, \end{array}$$

(69) $$\begin{array}{cc} & L=\frac{1}{2}\int_{\Delta^{d}}\frac{\nabla \hat{G}\left(x\right)}{\hat{G}\left(x\right)}c\left(x\right)\frac{\nabla \hat{G}\left(x\right)}{\hat{G}\left(x\right)}\varrho \left(dx\right)<\infty \end{array}$$

for some concave function $\hat{G}\in C^{2}\left({\bar{\Delta }}^{d}\right)$. Then we have P‐a.s.

(70) $$\begin{array}{c} \lim_{M\to \infty }\lim_{T\to \infty }\frac{1}{T}\log\left(V_{T}^{*,M,\frac{1}{M}}\right)=\lim_{T\to \infty }\frac{1}{T}\log\left(V_{T}\left(\nu \right)\right)=\lim_{T\to \infty }\frac{1}{T}\log\left({\hat{V}}_{T}\right)=L. \end{array}$$

Note first that *L* is well‐defined due to (69). Furthermore, note that for every ε>0, there exists some M>0 and some function $G\in G^{M,\frac{1}{M}}$ such that

$$\begin{array}{c} \lim_{T\to \infty }\frac{1}{T}\log\left(V_{T}^{G}\right)\geq \lim_{T\to \infty }\frac{1}{T}\log\left({\hat{V}}_{T}\right)+\varepsilon . \end{array}$$

Indeed this simply follows from continuity of $G\mapsto V^{G}$ as asserted in Lemma 4.4 and by choosing $G\in G^{M,\frac{1}{M}}$ close enough with respect to the ${∥\cdot ∥}_{C^{2,0}}$ to the optimizing function $\hat{G}\in C^{2}\left({\bar{\Delta }}^{d}\right)$ whose generated portfolio yields $\hat{V}$ due to (68) and Proposition 4.7. By Theorem 4.12, we can therefore conclude (following the proof of Corollary 3.11) that

(71) $$\begin{array}{c} \lim_{M\to \infty }\underset{T\to \infty }{lim inf}\frac{1}{T}\log\left(V_{T}^{*,M,\frac{1}{M}}\right)=\underset{T\to \infty }{lim inf}\frac{1}{T}\log\left(V_{T}\left(\nu \right)\right)=\lim_{T\to \infty }\frac{1}{T}\log\left({\hat{V}}_{T}\right)=L \end{array}$$

holds true. As Theorem 4.11 implies that

$$\underset{T\to \infty }{lim sup}\frac{1}{T}\log V_{T}\left(\nu \right)=\lim_{M\to \infty }\underset{T\to \infty }{lim sup}\frac{1}{T}\log V_{T}^{*,M,\frac{1}{M}},$$

the assertion is proved if

(72) $$\begin{array}{c} \underset{T\to \infty }{lim sup}\frac{1}{T}\left(\log V_{T}\left(\nu \right)-\log{\hat{V}}_{T}\right)=\underset{T\to \infty }{lim sup}\frac{1}{T}\log\left(\frac{V_{T}\left(\nu \right)}{{\hat{V}}_{T}}\right)=0,\;P\text{-a.s.} \end{array}$$

By the considerations of Section 4.2.3 (see also Becherer, 2001, proposition 4.3), it follows that ${\left(\frac{V_{t}\left(\nu \right)}{{\hat{V}}_{t}}\right)}_{t\geq 0}$ is a nonnegative supermartingale. It converges P‐a.s. to a finite limit as t→∞. This in turn implies (72) and proves the statement. □

Finally, a similar result can be obtained by restricting the log‐optimal portfolio to the class of *C* ^2^‐functionally generated portfolios without imposing the drift condition in Proposition 4.7. We denote by $V_{T}^{\mathrm{fun}}$ the wealth process of the log‐optimal portfolio among concave *C* ^2^‐functionally generated portfolios, that is, $\pi^{\mathrm{fun}}$ is defined as in (60), however by taking the arg max over all concave *C* ^2^‐functionally generated portfolios.

Corollary 4.14 Let ${\left(\mu_{t}\right)}_{t\geq 0}$ be a stochastic process of form (47) satisfying Assumption 4.8. Moreover, suppose that (61) and (62) hold true. Then

(73) $$\begin{array}{c} \lim_{M\to \infty }\underset{T\to \infty }{lim inf}\frac{1}{T}\log V_{T}^{*,M,\frac{1}{M}}=\underset{T\to \infty }{lim inf}\frac{1}{T}\log V_{T}\left(\nu \right)=\lim_{T\to \infty }\frac{1}{T}\log V_{T}^{\mathrm{fun}},\;P-a.s. \end{array}$$

The proof is the same as the first part of Corollary 4.13 up to (71). Note that we cannot get rid of the lim inf because the supermartingale argument from the proof of Corollary 4.13 does not hold. □

## ENDNOTES

1 Henceforth, we only use the terminology log‐optimal portfolio.

2 Here, the portfolio weight $\pi_{t}$ is chosen at time t−1 and is used over the time interval [t−1,t].

3 We are grateful to one of anonymous referees for pointing this out.

4 Later we will use *V* to denote instead the *relative* wealth of the portfolio.

5 Here, it is assumed implicitly that the stocks do not pay dividends. This assumption is common in universal and SPT and allows us to focus on the main ideas.

6 A comparison between the log‐optimal portfolio and Cover's universal portfolio is studied in Ichiba et al. (2011).

7 To simplify the notations, we will suppress ω.

8 By a slight abuse of notation, we here write $\hat{\pi }$ although we do not restrict to long‐only portfolios as in Section 2.3.

## APPENDIX 1 PROOFS OF CERTAIN RESULTS AND LEMMAS

Proof of Theorem 2.1 Fix T>0 and the trajectory $s={\left(s_{t}^{1},\cdots ,s_{t}^{d}\right)}_{t=0}^{T}\in {\left(R^{d}\right)}^{T+1}$. For fixed *s* the function $b\mapsto V_{T}\left(b\right)\left(s\right)$ is continuous on ${\bar{\Delta }}^{d}$. Hence there is $\bar{b}=\bar{b}\left(s\right)\in {\bar{\Delta }}^{d}$ such that

(A1) $$\begin{array}{c} V_{T}^{*}\left(s\right)=V_{T}\left(\bar{b}\right)\left(s\right). \end{array}$$

In fact, condition (9) implies that the sequence of functions ${\left(b\mapsto \frac{1}{T}\log V_{T}\left(b\right)\right)}_{T=1}^{\infty }$ is Lipschitz on ${\bar{\Delta }}^{d}$, uniformly in T∈N and *s* satisfying (9) for some fixed constants C>c>0. Indeed, consider the distance on ${\bar{\Delta }}^{d}$ defined by $∥b-\tilde{b}{∥}_{1}=\sum_{j=1}^{d}\left|b^{j}-{\tilde{b}}^{j}\right|$. Then we may estimate

$$\begin{array}{c} \left|\frac{1}{T}\log V_{T}\left(b\right)-\frac{1}{T}\log V_{T}\left(\tilde{b}\right)\right|\leq \left(\log\left(C\right)-\log\left(c\right)\right){∥b-\tilde{b}∥}_{1}. \end{array}$$

For ε>0, we may therefore define $\delta :=\frac{c\varepsilon }{C}>0$ such that, for every δ‐neighborhood $U(\bar{b})$ around any $\bar{b}\in {\bar{\Delta }}^{d}$ we have

$$\begin{array}{c} \frac{1}{T}\log V_{T}\left(b\right)\geq \frac{1}{T}\log V_{T}\left(\bar{b}\right)-\varepsilon \end{array}$$

for every $b\in U(\bar{b})$. If the probability measure ν has full support, we also may find η=η(ε,c,C)>0 such that each such δ‐neighborhood $U(\bar{b})$, where $\bar{b}$ runs through ${\bar{\Delta }}^{d}$, satisfies $\nu (U\left(\bar{b}\right))>\eta$. Using (A1) we therefore may conclude, similarly as in (A8), that (8) holds true, uniformly in $s={\left(s_{t}^{1},\cdots ,s_{t}^{d}\right)}_{t=0}^{\infty }$ satisfying (9) for some fixed constants C>c>0.□

Proof of Lemma 3.8 Let $\hat{\pi }:\Delta^{d}\to {\bar{\Delta }}^{d}$ be the optimizer of (18) and define, for 0<ε<1,

$$\pi_{\varepsilon }=\left(1-\varepsilon \right)\hat{\pi }+\varepsilon \left(\frac{1}{d},\cdots ,\frac{1}{d}\right).$$

Note that $\pi_{\varepsilon }$ takes values in ${\bar{\Delta }}_{\varepsilon }^{d}$ (see Definition 3.1), which is crucial for the subsequent arguments and the reason why we do not directly work with $\hat{\pi }$. Also note that, for $p\in {\bar{\Delta }}_{\varepsilon }^{d}$, we have

(A2) $$\begin{array}{c} 〈p,\frac{y}{x}〉=\sum_{j=1}^{d}p^{j}\frac{y^{j}}{x^{j}}\geq \frac{\varepsilon }{d} \end{array}$$

for $x,y\in \Delta^{d}$, as at least one of the terms $\frac{y^{j}}{x^{j}}$ is greater than or equal to one. The average performance $L^{\pi_{\varepsilon }}$ defined via (22) for the portfolio map $\pi_{\varepsilon }$ is still almost as good as the optimal average performance $L\equiv L^{\hat{\pi }}$:

(A3) $$\begin{array}{ccc} L^{\pi_{\varepsilon }} & = & \int_{\Delta^{d}}\left[\int_{\Delta^{d}}\log\left(〈\pi_{\varepsilon }\left(x\right),\frac{y}{x}〉\right)\varrho \left(x,dy\right)\right]d\varrho \left(x\right)\\ & \geq & \int_{\Delta^{d}}\left[\int_{\Delta^{d}}\log\left(\left(1-\varepsilon \right)〈\hat{\pi }\left(x\right),\frac{y}{x}〉\right)\varrho \left(x,dy\right)\right]d\varrho \left(x\right)\\ & \geq & L+\log(1-\varepsilon ). \end{array}$$

To approximate $\pi_{\varepsilon }$ by a Lipschitz function $\pi_{Lip}$ taking its values in ${\bar{\Delta }}_{\varepsilon }^{d}$, we need some preparation. By Assumption 3.6, we can find δ>0 such that, for $A\subseteq \Delta^{d}$,

(A4) $$\int_{A}\left[\int_{\Delta^{d}}\left(\log\left(\frac{\varepsilon }{d}\right)-\log\left(〈\pi_{\varepsilon }\left(x\right),\frac{y}{x}〉\right)\right)\varrho \left(x,dy\right)\right]d\varrho \left(x\right)>-\varepsilon$$

provided that ϱ[A]<δ. In particular, we may find η>0 such that

(A5) $$\int_{\Delta^{d}\setminus {\bar{\Delta }}_{\eta }^{d}}\left[\int_{\Delta^{d}}\left(\log\left(\frac{\varepsilon }{d}\right)-\log\left(〈\pi_{\varepsilon }\left(x\right),\frac{y}{x}〉\right)\right)d\varrho \left(x,y\right)\right]d\varrho \left(x\right)>-\varepsilon .$$

Now we find a Lipschitz function $\pi_{Lip}:\Delta^{d}\to {\bar{\Delta }}_{\varepsilon }^{d}$ such that

(A6) $$∥\pi_{Lip}\left(x\right)-\pi_{\varepsilon }{\left(x\right)∥}_{1}=\sum_{j=1}^{d}\left|\pi_{Lip}{\left(x\right)}^{j}-\pi_{\varepsilon }{\left(x\right)}^{j}\right|<\eta \varepsilon^{2}$$

for all $x\in \Delta^{d}\setminus A$, where the exceptional set *A* satisfies ϱ[A]<δ. Indeed, the functions from $R^{d}\to {\bar{\Delta }}_{\varepsilon }^{d}$ which are continuously differentiable in a neighborhood of $\Delta^{d}$ are dense with respect to the $L^{1}\left(R^{d},\varrho ;R^{d}\right)$‐norm. Let *M* be a Lipschitz constant for $\pi_{Lip}$ such that $M^{-1}\leq \varepsilon$. To estimate $L^{\pi_{Lip}}-L^{\pi_{\varepsilon }}$ we argue separately on the sets $\Delta^{d}\setminus {\bar{\Delta }}_{\eta }^{d},A\cap {\bar{\Delta }}_{\eta }^{d}$ and ${\bar{\Delta }}_{\eta }^{d}\setminus A$. To start with the latter set note that, for $x\in {\bar{\Delta }}_{\eta }^{d}$ and $y\in \Delta^{d}$ we have that the function

$$p\mapsto 〈p,\frac{y}{x}〉=\sum_{i=1}^{n}p^{j}\frac{y^{j}}{x^{j}},\;p\in {\bar{\Delta }}^{d}$$

is Lipschitz on ${\bar{\Delta }}^{d}$ with Lipschitz constant bounded by ${\left(\frac{\eta }{d}\right)}^{-1}$. From (A6), we get

(A7) $$\begin{array}{c} \int_{{\bar{\Delta }}_{\eta }^{d}\setminus A}\left[\int_{\Delta^{d}}\left(\log\left(〈\pi_{Lip}\left(x\right),\frac{y}{x}〉\right)-\log\left(〈\pi_{\varepsilon }\left(x\right),\frac{y}{x}〉\right)d\varrho \left(x,y\right)\right]d\varrho \left(x\right)\right.\\ \geq -\left(\eta \cdot \varepsilon^{2}\right){\left(\frac{\eta }{d}\right)}^{-1}{\left(\frac{\varepsilon }{d}\right)}^{-1}\geq -d^{2}\varepsilon . \end{array}$$

The term ${\left(\frac{\varepsilon }{d}\right)}^{-1}$ above comes from the fact that $\left\langle \pi_{Lip}\left(x\right),\frac{y}{x}\right\rangle$ as well as $\left\langle \pi_{\varepsilon }\left(x\right),\frac{y}{x}\right\rangle$ takes values in $[\frac{\varepsilon }{d},\infty [$ and the function z↦log(z) is Lipschitz on this set with constant ${\left(\frac{\varepsilon }{d}\right)}^{-1}$. As regards the set $A\cap {\bar{\Delta }}_{\eta }^{d}$ we obtain from (A2) and (A4) the estimate

(A8) $$\int_{A\cap {\bar{\Delta }}_{\eta }^{d}}\left[\int_{\Delta^{d}}\left(\log\left(〈\pi_{Lip}\left(x\right),\frac{y}{x}〉\right)-\log\left(〈\pi_{\varepsilon }\left(x\right),\frac{y}{x}〉\right)d\varrho \left(x,y\right)\right]d\varrho \left(x\right)\geq -\varepsilon \right.$$

and a similar estimate holds true for the set $\Delta^{d}\setminus {\bar{\Delta }}_{\eta }^{d}$ by (A5). Hence, we obtain from (A3), (A7), and (A8)

$$L^{\pi_{Lip}}\geq L+\log\left(1-\varepsilon \right)-d^{2}\varepsilon -2\varepsilon .$$

As ε>0 is arbitrary, we have proved Lemma 3.8. □

Proof of Lemma 4.1 This follows from the fact that the embedding from $C^{2,\alpha }\left({\bar{\Delta }}^{d}\right)\to C^{2,\alpha^{\prime }}\left({\bar{\Delta }}^{d}\right)$ is compact for $\alpha^{\prime }<\alpha$ (see, e.g., Dobrowolski, 2010, satz 2.42). This means in particular that any bounded set in $C^{2,\alpha }\left({\bar{\Delta }}^{d}\right)$ is totally bounded in $C^{2,0}\left({\bar{\Delta }}^{d}\right)$, thus relatively compact. To prove compactness it thus suffices to prove that $G^{M,\alpha }$ is closed. Take a sequence $G^{n}\in G^{M,\alpha }$ converging to *G* with respect to the ${∥\cdot ∥}_{C^{2,0}}$ norm. Then, we can estimate ${∥G∥}_{C^{2,\alpha }}$ by

$$\begin{array}{cc} & {∥G∥}_{C^{2,\alpha }}={∥G∥}_{C^{2,0}}+\max_{|k|=2}\underset{x\neq y}{\sup}\frac{|D^{k}G\left(x\right)-D^{k}G\left(y\right)|}{{∥x-y∥}^{\alpha }}\\ & \leq ∥G-G^{n}{∥}_{C^{2,0}}+{∥G^{n}∥}_{C^{2,0}}\\ & \;+\max_{|k|=2}\underset{x\neq y}{\sup}\frac{|D^{k}G\left(x\right)-D^{k}G^{n}\left(x\right)|+|D^{k}G^{n}\left(x\right)-D^{k}G^{n}\left(y\right)|+|D^{k}G^{n}\left(y\right)-D^{k}G\left(y\right)|}{{∥x-y∥}^{\alpha }} \end{array}$$

for any n∈N. Letting n→∞ and using the fact that $∥G^{n}{-G∥}_{C^{2,0}}\to 0$ yields ${∥G∥}_{C^{2,\alpha }}\leq M$. Similarly, we obtain $G\geq \frac{1}{M}$. This together with the fact that *G* is concave as a limit of concave functions proves $G\in G^{M,\alpha }$ and thus in turn compactness of $G^{M,\alpha }$ with respect to ${∥\cdot ∥}_{C^{2,0}}$. □

Proof of Lemma 4.4 For $G,\tilde{G}\in G^{M,\alpha }$, we have

$$\begin{array}{cc} & \log\left(V_{T}^{G}\right)-\log\left(V_{T}^{\tilde{G}}\right)=\log\left(G\left(\mu_{T}\right)\right)-\log\left(\tilde{G}\left(\mu_{T}\right)\right)-\left(\log\left(G\left(\mu_{0}\right)\right)-\log\left(\tilde{G}\left(\mu_{0}\right)\right)\right)\\ & \;\;-\int_{0}^{T}\left(\sum_{i,j}\frac{D^{ij}G\left(\mu_{t}\right)}{2G(\mu_{t})}-\frac{D^{ij}\tilde{G}\left(\mu_{t}\right)}{2\tilde{G}\left(\mu_{t}\right)}\right)d{\left[\mu^{i},\mu^{j}\right]}_{t}\\ & \;=\log\left(G\left(\mu_{T}\right)\right)-\log\left(\tilde{G}\left(\mu_{T}\right)\right)-\left(\log\left(G\left(\mu_{0}\right)\right)-\log\left(\tilde{G}\left(\mu_{0}\right)\right)\right)\\ & \;\;-\sum_{i,j}\int_{0}^{T}\left(\frac{D^{ij}G\left(\mu_{t}\right)-D^{ij}\tilde{G}\left(\mu_{t}\right)}{2G(\mu_{t})}+\frac{D^{ij}\tilde{G}\left(\mu_{t}\right)\left(\tilde{G}\left(\mu_{t}\right)-G\left(\mu_{t}\right)\right)}{2\tilde{G}\left(\mu_{t}\right)G\left(\mu_{t}\right)}\right)d{\left[\mu^{i},\mu^{j}\right]}_{t}. \end{array}$$

Hence, using the fact that $∥\tilde{G}{∥}_{C^{2,0}}\leq M$ as well as $G\geq \frac{1}{M}$ and $\tilde{G}\geq \frac{1}{M}$ and that z↦log(z) is Lipschitz continuous on $\left[\frac{1}{M},\infty \right)$ with constant *M*, we obtain the estimate

(A9) $$\begin{array}{ccc} & & \left|\log\left(V_{T}^{G}\right)-\log\left(V_{T}^{\tilde{G}}\right)\right|\leq 2M{∥G-\tilde{G}∥}_{C^{2,0}}\\ & & \;+\left(\frac{M}{2}d^{2}∥G-\tilde{G}{∥}_{C^{2,0}}+\frac{M^{3}}{2}d^{2}{∥G-\tilde{G}∥}_{C^{2,0}}\right)\max_{i}{\left[\mu^{i},\mu^{i}\right]}_{T}. \end{array}$$

This proves the asserted continuity. □
